# The phosphatase and tensin homolog gene inserted between NP and P gene of recombinant New castle disease virus oncolytic effect test to glioblastoma cell and xenograft mouse model

**DOI:** 10.1186/s12985-022-01746-w

**Published:** 2022-01-29

**Authors:** Sung Hoon Jang, Bo-Kyoung Jung, Yong Hee An, Hyun Jang

**Affiliations:** 1 Libentech Co. LTD, C-722 Daedeok BIZ Center, Techno 4-ro, 17 Yuseong-gu, Daejeon, 34013 Republic of Korea; 2 Department of Biological Sciences, College of Natural Sciences, Inha University, Incheon, 22212 Republic of Korea

**Keywords:** New castle disease virus, Phosphate and tensin homolog, Glioblastoma, Oncolysis, Tumor suppression

## Abstract

**Background:**

Glioblastoma is one of the most serious brain cancer. Previous studies have demonstrated that PTEN function disorder affects the causing and exacerbation of glioblastoma. Newcastle disease virus (NDV) has been studied as a cancer virotherapeutics. In this study, PTEN gene was delivered to glioblastoma by recombinant NDV (rNDV) and translated into protein at the cytoplasm of the glioblastoma.

**Methods:**

We did comparison tests PTEN protein expression efficiency and oncolytic effect depend on the PTEN gene insertion site at the between NP and P genes and the between P and M gene. PTEN protein mRNA transcription, translation in glioblastoma cell, and functional PTEN protein effect of the rNDV in vitro and in vivo test performed using western blotting, RT-qPCR, MTT assay, and Glioblastoma xenograft animal model test.

**Results:**

The result of this study demonstrates that rNDV-PTEN kills glioblastoma cells and reduces cancer tissue better than rNDV without the PTEN gene. In molecular immunological and cytological assays, PTEN expression level was high at located in the between NP and P gene, and PTEN gene was successfully delivered to the glioblastoma cell using rNDV and PTEN gene translated to functional protein and inhibits hTERT and AKT gene.

**Conclusions:**

PTEN gene enhances the oncolytic effect of the rNDV. And our study demonstrated that NP and P gene site is better than P and M gene site which is commonly and conventionally used. PTEN gene containing rNDV is a good candidate virotherapeutics for glioblastoma.

**Supplementary Information:**

The online version contains supplementary material available at 10.1186/s12985-022-01746-w.

## Introduction

Glioblastoma or glioma, one of the most serious brain cancer originates from glial cells that is a fatal tumor in the human central nervous system [1, 2]. The routine treatments for glioblastoma patients are surgical operations combined with radiotherapy, chemotherapy, and other effective treatments [3, 4]. Commonly observed symptoms of glioblastoma are persistent headaches, vomiting, double or blurred vision, loss of appetite, change in mood and personality, seizures, cerebral hemorrhage, and symptoms related to specific parts of the brain suspected of having glioblastoma [5]. According to case studies show 75% of all malignant brain tumors are glioblastoma [6]. Malignant grades of glioblastoma are diverse in appearance depending on the variations in infiltrative growth [7–9]. Thus, while glioblastoma is of clinical interest, developing effective treatments therapeutics for patients remains unsolved.

Oncolytic virotherapy is a perspective form of new biological therapeutics for various cancer treatments. The current oncolytic viruses are constructed using cancer cell killing or immune response electing gene to increase the efficacy of the virotherapeutics. Other virotherapeutics using intrinsic oncolytic virus or cancer cell killing effective gene containing intrinsic oncolytic virus were tried to using cancer treatment. Several results show utilizes viral intrinsic oncolytic properties and specific genes more effectively destroy malignant tumor cells than utilize oncolytic virus or specific gene [10]. Since the 1950s several research has been tried using NDV for cancer treatment [11]. NDV could be developed as a perspective anticancer virotherapeutics for cancer patients but remain how could increase the oncolytic effect of lentogenic strain using safety-guarantied technology. The research about the basic mechanism of oncolytic effect has been studied by the velogenic NDV strains and that show strong tumor suppression result from many brain cancer patients during the clinical test [12]. But velogenic strain has safety issue as a pathogen of the avian infectious disease, the lentogenic strain used instead of velogenic strain. Intrinsic oncolytic effect of the lentogenic strain less than that of the velogenic strain, several studies were performed increase oncolytic effect using several tumor suppression gene containing rNDV has been constructed, which was show cancer cell killing and tumor suppression effect to various cancer. NDV can not propagate in the normal cell but in cancer. Because in cancer cell IFN-α mediated innate immune response is not elect by virus infection. Most of cancer cell do not produce IFN-α and not properly work the cytokine-producing signal pathway [13–16]. Newcastle disease virus (NDV) is one of the old history cancer viral therapeutics. In the 1950s velogenic strain of NDV has been used to cancer-killing effect test even some of the oncolytic effects test was doing in Human patients. Several research and clinical studied show perspective to cancer treatment because NDV does not infect mammalian and no side effect by repeat inoculation and cancer cell killing effect and tumor suppression effect also good to patients who participated in clinical trial survive more than five years [17]. But NDV velogenic strain is a serious pathogen of poultry disease it can be a big damage to the poultry industry. So, this is one of the obstacles of NDV develop viral cancer therapeutics. In the 2000s many scientists start using a lentogenic strain of NDV used to developing as an oncolytic virus. But this lentogenic strain also has defects not strong cancer cell killing effect or tumor suppress effect compare with velogenic strain and the reason is lentogenic strain show lysogenic virus-like does not occur reinfection to neighbor cancer cell after the first infection. To overcome this defect, several cancer cell killing and tumor suppress gene containing recombinant NDV virus has been constructed and used to test anticancer effect test include clinical trial. This kind of progress successfully makes good results comparing to just attenuated NDV vaccine strain used previous test [18]. One more advantage of NDV compare with the currently used viral vector, NDV can pass the brain-blood barrier (BBB) and be transmitted to the human brain. It is very suitable as a viral vector for glioblastoma treatment [19]. Now increasing NDV oncolytic effect several related area researches were performed such as the most efficient foreign gene insertion site and expression of a functional molecule using recombinant NDV. A most important study is the development of NDV using cancer therapeutics that NDV or recombinant NDV was how they get the selectively infect to a specific cancer cell and not infected to a normal cell.

PTEN protein is a well-known tumor suppressor protein that acts as a phosphatase enzyme that catalysis the dephosphorylation of the 3′ phosphate of the inositol ring which is in PIP3 and makes the biphosphate product PIP2. This dephosphorylation act a key role in regulating cellular behaviors such as migration, survival, and cell growth. Deletions and mutations of PTEN inactivate its enzymatic activity during tumor development leading to reduced cell death and increased cell proliferation. Frequent genetic disorder of PTEN occurs in prostate cancer, endometrial cancer, and glioblastoma; and decreased expression is found in a lot of other tumor types such as breast and lung cancer. Current studies show about 50% of GBMs have a somatic change in the phosphatidylinositol 3-OH kinase pathway [20, 21]. Weak protein phosphatase activity is also important to tumor suppression. Protein phosphatase activity of PTEN may be involved in cell cycle regulation, preventing cells from dividing and growing too promptly.

In the present study, newly constructed recombinant NDV(rNDV) based on the VG/GA lentogenic strain [22] which has the PTEN gene and Kozak sequence in the rNDV genome and tests the oncolytic effect in vitro and in vivo.

## Materials and methods

### cDNA construction of NDV containing PTEN CDS

**Fig. 1:**
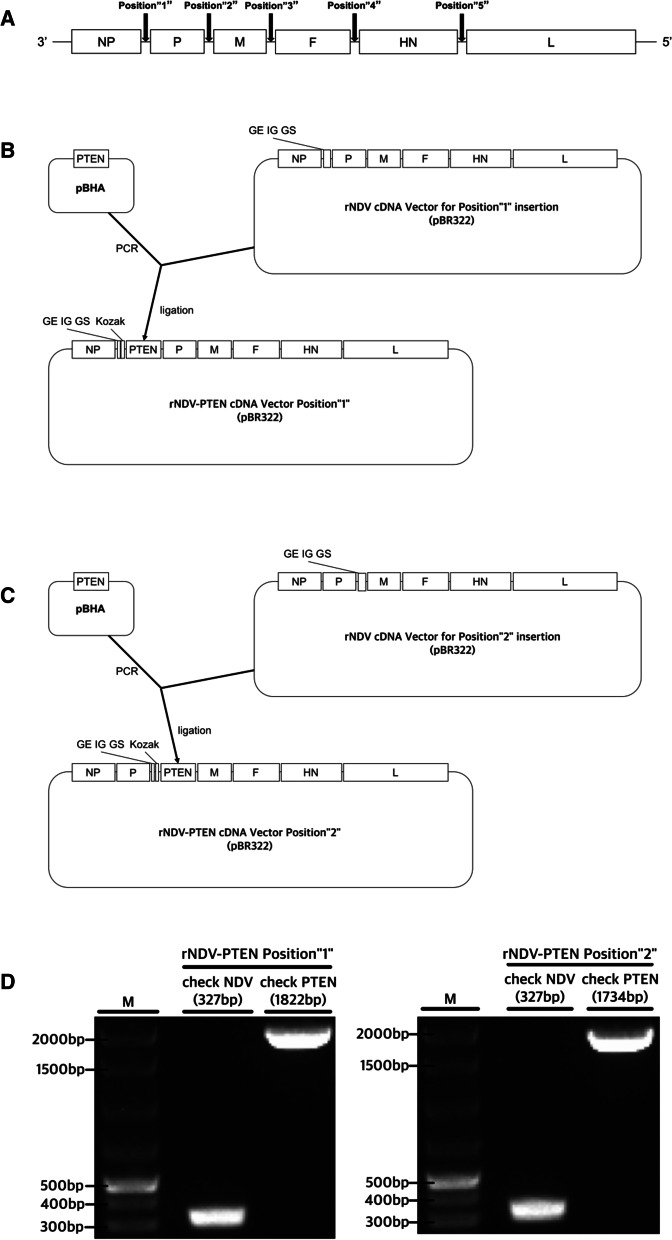
Construction of recombinant NDV. **A** Possible foreign gene insertion sites are marked in order. **B** Gene map of PTEN inserted rNDV Position “1”. **C** Gene map of PTEN inserted rNDV Position “2”. **D** Electrophoresis photograph to confirm the existence of NDV (L gene fragment) and inserted PTEN gene

The 1212 base pair DNA sequence encoding human PTEN CDS (NM_000314.8) was synthesized and provided from Bioneer (Daejeon, Republic of Korea). In this study, PTEN gene was inserted between NP and P genes or between P and M genes. Two sites are designated Position “1” (NP-P) and Position “2” (P-M) (Fig. 1A). Fse I enzyme sequence, Kozak sequence, and PTEN CDS for insert Position “1” were amplified by performing twice of polymerase chain reactions. Using Phusion Flash High-Fidelity PCR Master Mix (cat. No. F548S; Thermo Fisher Scientific, Inc., Waltham, MA, USA) and 4 primers (Table 1) of ‘Kozak PTEN for Position “1” (1st PCR)’, ‘Fse I Kozak PTEN for Position “1” (2nd PCR)’ polymerase chain reactions were performed according to the manufacturer’s instructions. PCR conditions for first PCR and second PCR were as follows: 1 cycle at 98 °C for 30 s, followed by 30 cycles at 98 °C for 10 s, 50 °C for 30 s, and 72 °C for 30 s. Not I enzyme sequence, Kozak sequence, and PTEN CDS for insert Position “2” were amplified by performing twice of polymerase chain reactions. Using Phusion Flash High-Fidelity PCR Master Mix (cat. No. F548S; Thermo Fisher Scientific, Inc., Waltham, MA, USA) and 4 primers (Table 1) of ‘Kozak PTEN for Position “2” (1st PCR)’ and ‘Not I Kozak PTEN for Position “2” (2nd PCR)’, polymerase chain reactions were performed according to the manufacturer’s instructions. PCR conditions for first PCR and second PCR were as follows: 1 cycle at 98 °C for 30 s, followed by 30 cycles at 98 °C for 10 s, 50 °C for 30 s, and 72 °C for 30 s. And PTEN CDS fragment was inserted into pBR322 [23] containing cDNA of NDV (Libentech Inc., Daejeon, Republic of Korea). Subcloning was performed by Fse I restriction enzyme (cat. no. R0558S; New England BioLabs, Inc., Ipswich, MA, USA), Not I restriction enzyme (cat. No. R0189S; New England BioLabs, Inc., Ipswich, MA, USA) and T4 DNA ligase (cat. No. M0202S; New England BioLabs, Inc., Ipswich, MA, USA). Agarose gel (1%) electrophoresis and Molecular Imager® Gel Doc™ XR + System (cat. No. 1708195EDU, Bio-Rad, Hercules, CA, USA) are used for confirming the PCR product and products of ligation. rNDV-PTEN cDNA vectors were acquired. Transforming this cDNA rNDV-PTEN vectors into Top 10 *E. coli* by heatshock was performed. rNDV-PTEN cDNA vector transformed *E. coli* colony was inoculated into a liquid medium and cultured overnight at 150 rpm and 37 °C to obtain a large amount of rNDV-PTEN cDNA vectors. Sequencing was performed to confirm that the rNDV-PTEN cDNA vectors have intact PTEN CDS.

**Table 1:** Primers for cDNA construction of NDV containing PTEN CDS

Gene	Direction	Sequence (5′ → 3′)	Size (bp)
Kozak PTEN for Position “1” (1st PCR)	Forward	GCCACCATGACAGCCATCATCAAAG	1221
	Reverse	AGAAGCGGCCGCGGCCGGCCACCATGACAGCC	
Fse I Kozak PTEN for Position “1” (2nd PCR)	Forward	GCCTCAGACTTTTGTAATTTGTGTATG	1258
	Reverse	GTTGGACCTTGGTATGGCCGGCCTCAGACTTTTGTAATTTGTG	
Kozak PTEN for Position “2” (1st PCR)	Forward	GCCACCATGACAGCCATCATCAAAG	1221
	Reverse	CGCTCAGACTTTTGTAATTTGTGTA	
Not I Kozak PTEN for Position “2” (2nd PCR)	Forward	ATACGGGTAGAAGCGGCCGCCACCATGACAGCC	1254
	Reverse	TGGACCTTGGGCGGCCGCTCAGACTTTTGTAATTTG	

### Transfection and recovery of recombinant NDV

NP, P, and L genes which form the NDV transcriptase complex were cloned into a pBR322 vector and used as a helper plasmid (pBR322-NP, pBR322-P, pBR322-L). HEp-2 cells were prepared in a 6-well plate at 5 × 10^5^ cells/well. After 24 h of culture, Vaccinia virus expressing T7 RNA polymerase infected HEp-2 cells (MOI 1). 5 μg of rNDV-PTEN cDNA vector was transfected into HEp-2 cells with 2.5 μg of NP helper plasmid, 1.5 μg of P helper plasmid, and 0.5 μg of L helper plasmid by Lipofectamine 3000 (Invitrogen; Thermo Fisher Scientific, Inc.) according to the manufacturer’s instructions. After 72 h of transfection, 100 μl HEp-2 cell supernatant was harvested and inoculated in the allantoic cavity of a 10-day-old specific-pathogen-free (SPF) embryonated chicken egg. After 96 h, the allantoic fluid was harvested. The harvested viruses were adapted to the Vero76 cell. Using TCID50 assay by Reed-Muench method, NDV titers and MOI are determined. In order to confirm the existence of the NDV and the inserted PTEN gene, reverse transcription PCR (RT-PCR) was performed with primers of ‘NDV check’ to amplify an rNDV L gene fragment, primers ‘NP-PTEN-P check’ to confirm inserted PTEN gene fragment in Position “1” and primers ‘P-PTEN-M check’ to confirm inserted PTEN gene fragment in Position “2”. (Table 2) (Fig. 1) RT-PCR conditions to amplify an rNDV L gene fragment and confirm inserted PTEN gene were as follows: 1 cycle at 42 °C for 1 h, 94 °C for 5 m, followed by 35 cycles at 94 °C for 60 s, 60 °C for 60 s, and 72 °C for 60 s. Sequencing was performed to confirm that the rescued rNDV-PTEN virus has proper PTEN CDS (Additional file 1: S1, S2). And another sequencing was performed whether the virus has intact PTEN CDS even when subcultured to Passage10 (Additional file 1: S3, S4).

**Table 2:** Primers to confirm NDV and PTEN CDS

Gene	Direction	Sequence (5′ → 3′)	Size (bp)
NDV check	Forward	CCACAATTCCAAGATAACCGGAG	327
	Reverse	GCTGCCACAATCAGATGCCTTTG	
NP-PTEN-P check	Forward	AACAGATCACAAGGGCAACCG	1822
	Reverse	TGGTTTTCCCTGGGCCGTAATT	
P-PTEN-M check	Forward	GGCAAGCGGGCCTGATATAGG	1734
	Reverse	CTTCCCGTCCCCTGTGTCTTG	

### Cell culture

The glioblastoma cell line T98G (cat. No. CRL-1690™, ATCC, Manassas, VA, USA) for in vitro/in vivo assay, the Vero76 cell line (cat. No. CCL-81™, ATCC, Manassas, VA, USA) for rNDV/rNDV-PTEN recovery, adaptation and production, and the normal cell line CCD-18Co (cat. No. CRL-1459™, ATCC, Manassas, VA, USA) for in vitro assay. The glioblastoma cell line T98G was cultured in Minimum Essential Medium (MEM, Lot.2177680, Gibco, Gaithersburg, MD, USA) including penicillin–streptomycin (Lot.15140112, Gibco, Gaithersburg, MD, USA) and 10% fetal bovine serum (Lot.10100139, Gibco, Gaithersburg, MD, USA) at 5% CO_2_, 37 °C. When the cells grow to form a 70–80% monolayer in the flask, the cells are maintained through subculture. The sprit ratio was 1:4 or 1:6, and the seeding density was 2–4 × 10^4^ cells/ml. The Vero76 cell line was cultured in Dulbecco’s Modified Eagle Medium (DMEM, Lot.2186826, Gibco, Gaithersburg, MD, USA) including penicillin–streptomycin (100 U/ml, HyClone, USA) and 10% fetal bovine serum (FBS, HyClone) at 5% CO_2_, 37 °C. When the cells grow to form a 70–80% monolayer in the flask, the cells are maintained through subculture. The sprit ratio was 1:8, and the seeding density was 1 × 10^4^ cells/ml. 0.25% trypsin and 0.02% EDTA solution were used for the cell dissociation process of the subculture. The CCD-18Co cell line was cultured in Dulbecco’s Modified Eagle Medium (DMEM, Lot.2186826, Gibco, Gaithersburg, MD, USA) including penicillin–streptomycin (100 U/ml, HyClone, USA) and 10% fetal bovine serum (FBS, HyClone) at 5% CO_2_, 37 °C. When the cells grow to form a 70–80% monolayer in the flask, the cells are maintained through subculture. The sprit ratio was 1:2 or 1:3, and the seeding density was 2 × 10^2^ cells/ml. 0.25% trypsin and 0.02% EDTA solution were used for the cell dissociation process of the subculture.

### Growth curve construction of rNDV and rNDV-PTEN

Vero76 cells were prepared in a 75 T flask at 4 × 10^6^ cells/flask. After 24 h of culture, Vero76 cells were infected by the rNDV-PTEN Position”1″ virus (MOI 0.1) and rNDV virus (MOI 0.1). Samplings were performed 8, 16, 24, 32, 40, 48, 56, 64, and 72 h after infection. Using TCID50 assay by Reed-Muench method, sample titers are determined.

### Real-time quantitative PCR

mRNA samples for evaluating PTEN, P-Akt, hTERT mRNA expression levels were extracted from rNDV, rNDV-PTEN Position “1” and rNDV-PTEN Position “2” infected T98G cells (MOI 1) and not infected T98G cells using AccuPrep^®^ Universal RNA Extraction Kit (Bioneer, Inc., Daejeon, Republic of Korea) according to the manufacturer’s instructions. RT-qPCR for mRNA from rNDV, rNDV-PTEN Position “1”, and rNDV-PTEN Position “2” infected T98G cells and not infected T98G cells was performed with primers and probes of ‘PTEN Real-Time’, ‘P-Akt Real-Time’ and ‘hTERT Real-Time’ (Table 3) to evaluate PTEN, P-Akt, and hTERT mRNA transcription level in T98G cells. Real-Time quantitative PCR was as follows: 1 cycle at 42 °C for 10 m, 95 °C for 2 m, followed by 40 cycles at 95 °C for 10 s, 59.8 °C for 60 s, and measure fluorescence level. CFX Connect™ Real-Time System (cat. No. 1855201 Bio-Rad, Hercules, CA, USA) was used. mRNA samples for evaluate Interferon-α mRNA expression levels were extracted from rNDV infected CCD-18Co cells (MOI 2), not infected CCD-18Co cells, rNDV infected T98G cells (MOI 2), and not infected T98G cells using AccuPrep® Universal RNA Extraction Kit (Bioneer, Inc., Daejeon, Republic of Korea) according to the manufacturer’s instructions. RT-qPCR for mRNA from CCD-18Co cells (MOI 2), not infected CCD-18Co cells, rNDV infected T98G cells (MOI 2), and not infected T98G cells was performed with primers and probe of ‘IFNA Real-Time’ (Table 3) to evaluate Interferon-α mRNA transcription level. Real-Time quantitative PCR was as follows: 1 cycle at 42 °C for 10 m, 95 °C for 2 m, followed by 40 cycles at 95 °C for 10 s, 58.3 °C for 60 s, and measure fluorescence level. CFX Connect™ Real-Time System (cat. No. 1855201 Bio-Rad, Hercules, CA, USA) was used. In all Real-Time quantitative PCR, beta-actin mRNA was used as an internal control.

**Table 3:** Primers for real-time quantitative PCR

Gene	Direction	Sequence (5′ → 3′)	Size(bp)
PTEN Real-Time	Forward	TCCCAGTCAGAGGCGCTATGT	152
	Reverse	GGCAGACCACAAACTGAGGA	
	Probe	[FAM]TGCAAGTTCCGCCACTGAACA[HEX]	–
P-Akt Real-Time	Forward	ACGGGCACATTAAGATCACAGACT	240
	Reverse	CCTCCATGAGGATGAGCTCAAAAAGC	
	Probe	CAATGACTACGGCCGTGCAGT[HEX]	–
hTERT Real-Time	Forward	CAGACGGTGTGCACCAACATCT	134
	Reverse	GTGTCAGAGATGACGCGCAGG	
	Probe	CAGCAAGTTTGGAAGAACCCCACA[HEX]	–
IFNA Real-Time	Forward	CAGTTCCAGAAGGCTCAAGCCA	304
	Reverse	TTTCTGCTCTGACAACCTCCCA	
	Probe	GAGAAGAAATACAGCCCTTGTGCC[HEX]	–
B-actin Real-Time	Forward	TGAAGTGTGACGTGGACATC	151
	Reverse	GGAGGAGCAATGATCTTGAT	
	Probe	ACGCCAACACAGTGCTGTCTG[HEX]	–

### Western blotting

T98G cells were prepared in a 175 T flask at 1 × 10^7^ cells/flask. After 24 h of culture, T98G cells were infected by the rNDV-PTEN Position “1” virus (MOI 1), rNDV-PTEN Position “2” virus (MOI 1), and rNDV virus (MOI 1). After 3, 6, 9 h of infection, protein extractions by 3 times of repeating freezing and thawing of the whole culture media (freeze–thaw method). Extracted protein solution mixed with sample buffer was denatured at 100 °C, 3 min. By using 12% SDS–polyacrylamide gel electrophoresis (PAGE), proteins are separated by size. Then, the protein was transferred to the PVDF membrane by a current of 25A for 15 min using Power Blotter (cat. No. PB0012, Thermo Fisher Scientific, Inc., Waltham, MA, USA). Blocking was performed by 1% BSA for 1 h. PTEN monoclonal antibody (1:1000 cat. No. ab32199 Abcam, Cambridge, United Kingdom), Phospho-AKT1 (Ser473) monoclonal antibody (1:1000 cat. No. 44-621G Invitrogen; Thermo Fisher Scientific, Inc.), Recombinant Anti-Telomerase reverse transcriptase monoclonal antibody (1: 1000 cat. No. ab32020 Abcam, Cambridge, United Kingdom) and beta Actin Monoclonal Antibody (1:2500 cat. No. MA1-140 Invitrogen; Thermo Fisher Scientific, Inc.) were used for the first probe. After 1 h incubation at RT, the membrane was washed 3 times and then anti-rabbit IgG goat horese peroxidase conjugate antibody (1:2500 Invitrogen; Thermo Fisher Scientific, Inc.) and anti-mouse IgG horese peroxidase conjugate antibody (1:2500 Invitrogen; Thermo Fisher Scientific, Inc.) were incubated for 1 h at RT. The membrane was washed 3 times and ECL (Enhanced chemiluminescence, Bio-Rad, Hercules, CA, USA) was added to the membrane. ChemiDoc™ MP Imaging System (cat. No. 17001402 Bio-Rad, Hercules, CA, USA) was used to visualize the protein. Based on the western blotting result, protein expression was quantified by ‘relative quantity’ of ‘Image lab’ program [24].

### MTT assay

An MTT assay is used to accurately measure live cells and evaluate changes in cell proliferation or death. 1 × 10^4^ T98G cells per well were plated in 96-well plates with Minimum Essential Medium (MEM, Lot.2177680, Gibco, Gaithersburg, MD, USA) including penicillin–streptomycin (100 U/ml, HyClone, USA) and 5% fetal bovine serum (FBS, HyClone) except one row(12 wells) for control. Four wells for phosphate-buffered saline (PBS), six wells for Dulbecco’s Modified Eagle Medium (DMEM, Gibco, USA) including penicillin–streptomycin (100 U/ml, HyClone, USA) and 10% fetal bovine serum (FBS, HyClone) were manufactured for control. After 24 h at 5% CO_2_, 37 °C for attach cells, rNDV, rNDV-PTEN Position “1” and rNDV-PTEN Position “2” were inoculated (MOI 0.1, 1, 2.5, 5). 20 μl of MTT solution of CellTiter 96® AQueous One Solution Cell Proliferation Assay (cat. No. G3582, Promega, Madison, WI, USA) was added to each well after 96 h of incubation. After adding of MTT solution, 1-h incubation at 5% CO_2_, 37 °C is performed. The absorption at 490 nm (OD490) was measured by iMark Microplate Reader (cat. No. 1681130EDU, Bio-Rad, Hercules, CA, USA). Cell viability was calculated as a percentage compared to the control.

### Animal analysis

Animal experiments were performed according to the protocols approved by the Institutional Animal Care and Use Committee of Libentech and all experiments conform to all relevant regulatory standards. A total of 20 SPF female BALB/c nude mice (5 weeks old; body weight, 14–19 g) were purchased from Japan SLC, Inc. (Hamamatsu, Japan). The environment for all mice was temperature-controlled at 22 ± 1°. A 12-h light/dark cycle was subjected to all mice. And all mice had ad libitum access to sterilized water and food. 6 × 10^6^ T98G cells in 100 μl of Minimum Essential Medium (MEM, Lot.2177680, Gibco, Gaithersburg, MD, USA) were mixed with 100 μl of Matrigel (cat. No. 354230, Corning, Corning, NY, USA) and then injected into the left flank of mice. When tumor volume reached 120–150 mm^3^ after 7 days after T98G cells inoculation, virotherapy was started. Mice with glioblastoma were randomly divided into 5 groups (n = 4). And 80 μl of 10^6.3^ TCID50/ml rNDV and rNDV-PTEN Position “1” were injected into the tumor-direct and tail vein. And 80 μl of dPBS was injected into the tumor-direct to the negative control group. Virotherapy was performed every day for a total of 7 days. Tumor volumes were every day calculated by using the formula: 4π/3 × (smallest diameter/2)^2^ × (largest diameter/2)^2^. Tumor size measurements were conducted 24 h post virus injection which is just before next injection of virus liquid.

### Statistical analysis

All data are presented as the mean ± the standard deviation of the means (SD), as indicated. Statistical comparisons were made by using Student’s t-test or one-way ANOVA with SPSS (Version 23, SPSS, Inc., Chicago, IL, USA). *p* Values < 0.05 were considered statistically significant.

## Results

### Growth kinetics and selective oncolytic effect of recombinant NDV infection to cancer cell

**Fig. 2:**
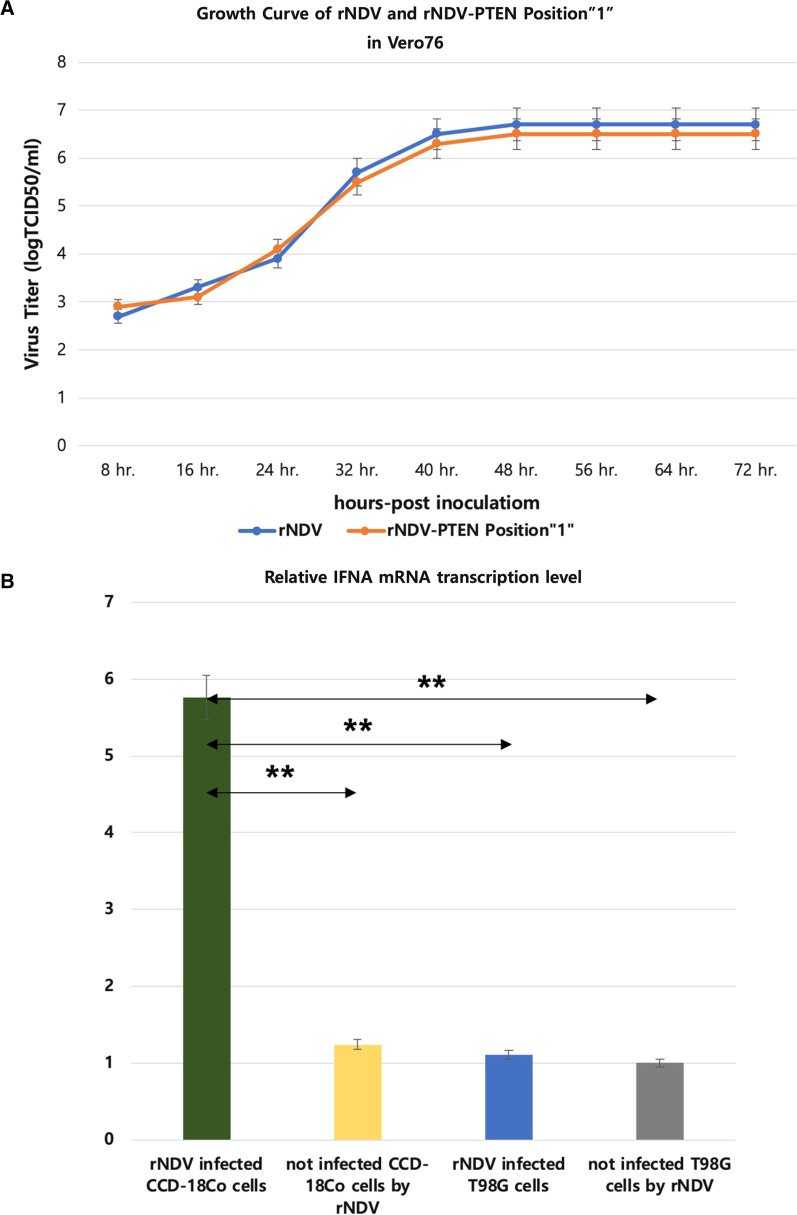
Growth kinetics and selective oncolytic effect of recombinant NDV infection to cancer cell. **A** Growth Curve of rNDV (control) and PTEN inserted rNDV Position “1” in Vero76. **B** Relative level of Interferon-α mRNA using Real-Time quantitative PCR in recombinant NDV (rNDV) infected normal cell (CCD18-Co), not infected normal cell (CCD18-Co), recombinant NDV (rNDV) infected glioblastoma cell (T98G), and not infected glioblastoma cell (T98G). Significant differences from control are indicated as **p < 0.01

To evaluate the rNDV replication efficiency, a growth dynamics curve in Vero76 cells was constructed (MOI 0.1) (Fig. 2A). The growth dynamics curve shows that the two rNDV viruses, one is having PTEN gene Position “1”, but both of the recombinant viruses’ growths have no significant difference. Both rNDV increase titer following the culture time and when 48 through 72 h post-infection show highest virus titer 10^6.7^ TCID_50_/ml rNDV and 10^6.5^ TCID_50_/ml rNDV-PTEN Position “1”.

Real-time quantitative PCR was used to assess interferon-α mRNA transcription levels (Fig. 2B). rNDV infected normal (CCD18-Co) cells have 4.64, 5.12, and 5.76 times higher interferon-α mRNA transcription levels than not infected normal (CCD18-Co) cells, rNDV infected glioblastoma (T98G) cells, and not infected glioblastoma (T98G) cells.

These results demonstrate that even when a foreign gene is inserted into rNDV, it proliferates as well as rNDV and the foreign gene is well expressed. And according to the expression of interferon-α, rNDV does not show apoptotic effect in normal cells but shows in glioblastoma cells.

### Effects of PTEN gene delivered by rNDV to apoptosis suppressor genes of T98G glioblastoma cells

**Fig. 3:**
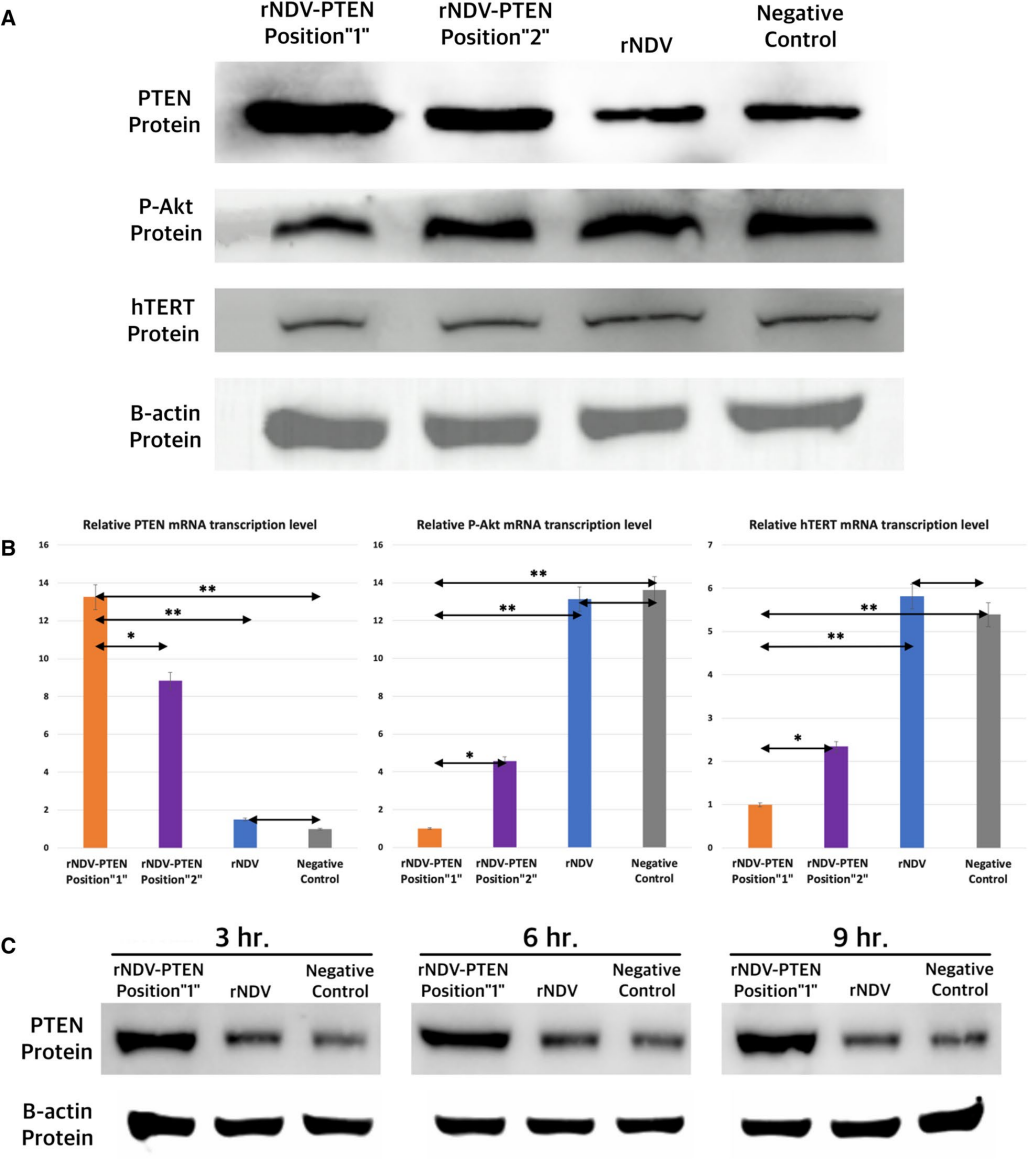
Effects of PTEN gene delivered by rNDV to apoptosis suppressor genes of T98G glioblastoma cells. **A** PTEN, P-Akt, and hTERT protein expression analysis using western blotting of PTEN inserted rNDV Position “1” infected glioblastoma cells, PTEN inserted rNDV Position “2” infected glioblastoma cells, rNDV infected glioblastoma cells, and not infected glioblastoma cells. B-actin protein western blotting is for loading control. **B** Relative level of PTEN, P-Akt and hTERT mRNA using Real-Time quantitative PCR in PTEN inserted rNDV Position “1" infected glioblastoma cell (T98G), PTEN inserted rNDV Position “2” infected glioblastoma cell (T98G), rNDV infected glioblastoma cell (T98G) and not infected glioblastoma cell (T98G) (negative control). Significant differences are indicated as *p < 0.05 and **p < 0.01. **C** PTEN protein expression analysis using western blotting of PTEN inserted rNDV Position”1″ infected glioblastoma cell (T98G), rNDV infected glioblastoma cell (T98G), and not infected glioblastoma cell (T98G) (negative control) at 3,6,9 h after infection

Western blotting was used for comparing PTEN protein, P-Akt protein, and hTERT protein expression between PTEN inserted rNDV Position “1” and PTEN inserted rNDV Position “2” (Fig. 3A) (MOI1). Both PTEN inserted rNDV infected glioblastoma cells showed increased PTEN protein expression than rNDV infected glioblastoma cells and not infected glioblastoma cells (negative control). PTEN protein expression level of PTEN inserted rNDV Position “1” infected glioblastoma cells was slightly higher than PTEN inserted rNDV Position “2” infected glioblastoma cells. Both PTEN inserted rNDV infected glioblastoma cells showed reduced P-Akt and hTERT protein expression than rNDV infected glioblastoma cells and not infected glioblastoma cells (negative control). P-Akt and hTERT protein expression level of PTEN inserted rNDV Position “1” infected glioblastoma cells were lower than PTEN inserted rNDV Position “2” infected glioblastoma cells.

Real-time quantitative PCR was used to assess PTEN, P-Akt, and hTERT mRNA transcription levels (Fig. 3B). rNDV-PTEN Position “1” infected glioblastoma cells have 1.5, 8, and 13 times higher PTEN mRNA transcription levels than rNDV-PTEN Position”2” infected, rNDV infected, and not infected glioblastoma cells. rNDV-PTEN Position “1” infected glioblastoma cells have 4, 13, and 13 times lower P-Akt mRNA transcription levels than rNDV-PTEN Position “2” infected, rNDV infected, and not infected glioblastoma cells. rNDV-PTEN Position “1” infected glioblastoma cells have 2, 5, and 5 times lower hTERT mRNA transcription levels than rNDV-PTEN Position “2” infected, rNDV infected, and not infected glioblastoma cells.

Western blotting was used for evaluating PTEN protein expression as time goes on (Fig. 3C). rNDV-PTEN Position “1” infected glioblastoma cells have significantly increased protein expression than rNDV infected glioblastoma cells and not infected glioblastoma cells in all times of this experiment. Quantification of Results of Western blotting was also performed (Additional file 1: S5).

These results demonstrate that rNDV-PTEN infected glioblastoma cells have increased PTEN expression and decreased P-Akt and hTERT activity due to increased PTEN expression. And PTEN inserted rNDV Position “1” is more effective than rNDV Position “2” to expression PTEN gene.

### PTEN gene containing rNDV induces growth inhibition and cell death of T98G glioblastoma cells

**Fig. 4:**
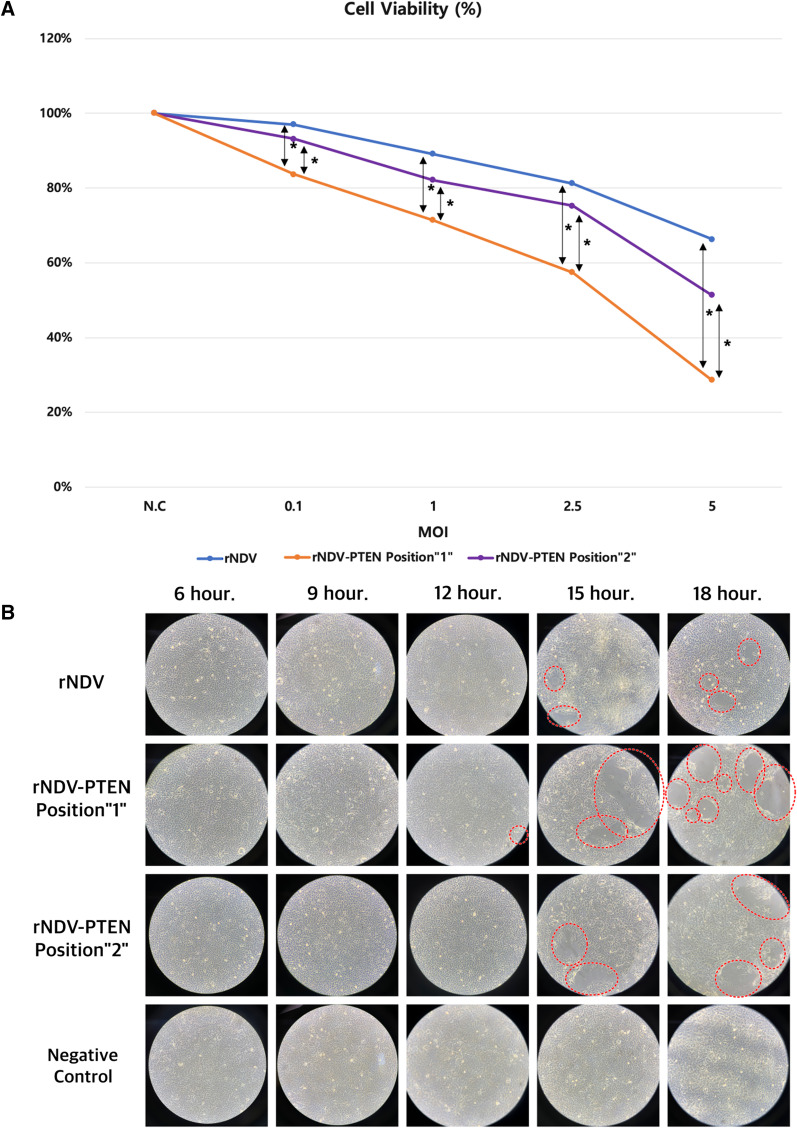
PTEN gene containing rNDV induces growth inhibition and cell death of T98G glioblastoma cells. **A** MTT assay of PTEN inserted rNDV Position “1” infected glioblastoma cell (T98G), PTEN inserted rNDV Position “2” infected glioblastoma cell (T98G), and rNDV infected glioblastoma cell (T98G). Significant differences are indicated as *p < 0.05. **B** Cytopathic effect (CPE) microscopic observation of glioblastoma cell (T98G) infected with PTEN inserted rNDV Position “1” (MOI 1), PTEN inserted rNDV Position “2” (MOI 1), rNDV (MOI 1), and not infected glioblastoma cell (T98G) (negative control). Scale bars, 100 μm

The effect of PTEN expression in glioblastoma cells was assessed. Using MTT assay, cell death rates were measured in glioblastoma cells infected with rNDV-PTEN Position “1”, rNDV-PTEN Position “2”, and rNDV(control) (Fig. 4A). In equal MOI, rNDV-PTEN Position “1” infected glioblastoma cells have 2.1–5.5 times higher apoptosis rate than rNDV (control) infected glioblastoma cells. And rNDV-PTEN Position “1” infected glioblastoma cells have 1.46–2.35 times higher apoptosis rate than rNDV-PTEN Position “2” infected glioblastoma cells in equal MOI. The lower MOI, the greater gap of apoptosis rate between rNDV-PTEN Position “1” and rNDV-PTEN Position “2” or rNDV infected glioblastoma cells.

Cytopathic effect(CPE) including rounding, vacuolation and syncytia formation was observed in T98G glioblastoma cells infected with rNDV-PTEN Position “1” (MOI 1), rNDV-PTEN Position “2” (MOI 1), rNDV (MOI 1), and no CPE at not infected glioblastoma cells (negative control) (Fig. 4B). Not infected glioblastoma cells (negative control) showed no CPE at all times. At 12 h after inoculation, the first CPE of rNDV-PTEN Position “1” infected glioblastoma cells was observed. And at 15 h, the first CPE of rNDV-PTEN Position “2” and rNDV infected glioblastoma cells was observed. At 15 h and 18 h after inoculation, more CPE was observed in rNDV-PTEN Position “1” infected glioblastoma cells than rNDV-PTEN Position “2” and rNDV infected glioblastoma cells.

These results demonstrate that rNDV-PTEN inhibits glioblastoma and removes tumor cells in vitro. And PTEN inserted rNDV Position “1” is more effective than rNDV Position “2” to remove tumor cells in vitro.

### PTEN gene containing rNDV induces size reduction of glioblastoma tumor on T98G cell planted xenograft mouse

**Fig. 5:**
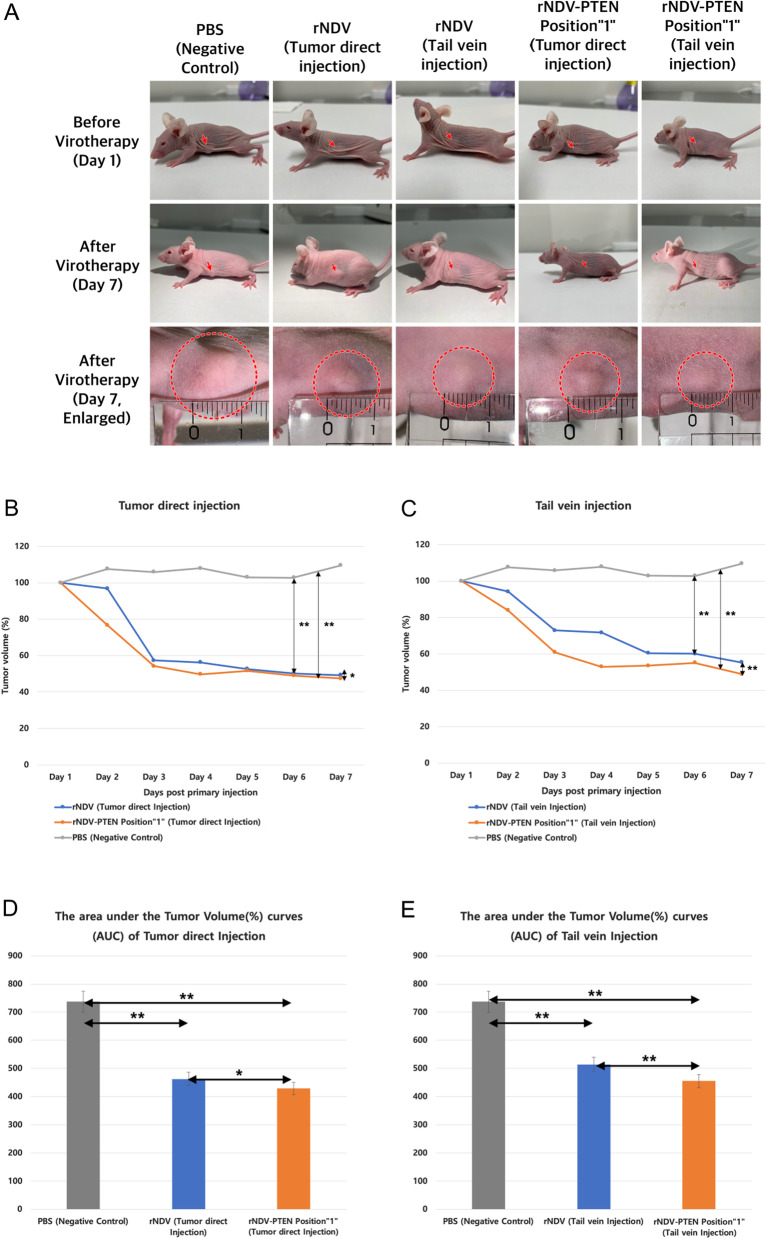
PTEN gene containing rNDV induces size reduction of glioblastoma tumor on T98G cell planted xenograft mouse. **A** Photograph of mice with glioblastoma treated PTEN inserted rNDV Position”1”, rNDV and PBS(negative control). **B** PTEN inserted rNDV Position “1”, rNDV and PBS(negative control) inoculated tumor volume (%) of mice with glioblastoma by tumor direct injection *p < 0.05 and **p < 0.01. **C** PTEN inserted Position “1”, rNDV and PBS (negative control) inoculated tumor volume (%) of mice with glioblastoma by tail vein injection **p < 0.01. **D** The area under the Tumor Volume (%) curves of Tumor direct Injection *p < 0.05 and **p < 0.01. **E** The area under the Tumor Volume (%) curves of Tail vein Injection **p < 0.01

Using mice, in vivo assay has been performed (Fig. 5, Table 4). At all injection sites, the tumor repression effect of rNDV-PTEN Position “1” was better than that of rNDV. Tumor injection was more effective than tail vein injection to reduce the volume of the tumor. rNDV-PTEN Position “1” injected tumors showed a steep decrease of tumor volume on day 2 of virotherapy and rNDV injected tumors showed a steep decrease of tumor volume on day 3 of virotherapy. On all days of virotherapy, the volume (%) of rNDV-PTEN Position “1” injected tumors was always smaller than rNDV injected tumors. rNDV-PTEN Position “1” effectively reduced the volume of glioblastoma tumors compared to rNDV and PBS groups. Especially, the gaps of reducing the volume of glioblastoma tumors between rNDV-PTEN Position “1” groups and rNDV groups or PBS groups were larger in tail vein injection. Orthotropic glioblastoma model in vivo test was also performed (Additional file 1: S6).

**Table 4:** Tumor volume (%) of mice with glioblastoma rNDV- PTEN, rNDV, and PBS (negative control)

Tumor volume(%)	Day 1	Day 2	Day 3	Day 4	Day 5	Day 6	Day 7
PBS (negative control)	100	107	105	107	102	102	109
rNDV (tumor direct Injection)	100	96	57	56	52	50	49
rNDV-PTEN (tumor direct Injection)	100	76	54	49	51	48	47
rNDV (tail vein Injection)	100	94	72	71	60	60	55
rNDV-PTEN (tail vein Injection)	100	83	60	52	53	55	48

This result demonstrates that rNDV-PTEN Position “1” is more effective than rNDV to reduce the size of glioblastoma in the xenograft mouse model.

## Discussion

The mutation of PTEN in various cancer is observed [25–28]. Therefore restore of PTEN expression in various cancer is important. Especially, PTEN mutation accounts for 34–44% of the cause of glioblastoma [29–31]. Elucidating the PTEN functions and the consequences of PTEN mutations allows the further understanding of cancer etiology and neoplasia. In glioblastoma, PTEN regulates glioblastoma oncogenesis by histone H3.3 and chromatin-associated complexes of DAXX [32] and mono-ubiquitination [33]. There is study indicated that PTEN inhibits lung cancer growth by promoting G0/G1 arrest and cell apoptosis, so rNDV-PTEN could be applied to lung cancer [34]. Immunotherapy which includes antigens on the cancer cell surface was used in various types of cancer [35–37]. Immunotherapy of tumors needs specific biomarkers of cancer cells such as specific receptors, recognition molecules, or antigen domains. Immunological response against occurred specifically against therapy and anti-neoplastic agents is effective [38–41]. As a result, restoration of normal PTEN expression in glioblastoma is very important in remove or therapy glioblastoma.

There have been disadvantages in glioblastoma virotherapeutics using virus vectors such as adenovirus, vaccinia virus, and adeno associated virus. In the case of the vaccinia virus, there is a limitation in repeated inoculation to humans because pre-existing immunity to the vaccinia virus is a problem in humans [42]. In the case of adenovirus or adeno-associated virus, glioblastoma removal or treatment is impossible because they cannot pass through the brain-blood barrier (BBB) and thus cannot access the glioblastoma [43]. However, NDV can be inoculated repeatedly because pre-existing immunity is not a prohibit cancer cell killing effect when inoculated into the human body, and NDV can pass through the brain-blood barrier (BBB), it is possible to infect glioblastoma cancer cells in the brain [44–47]. In addition, since NDV has an intrinsic oncolytic effect, it is possible to safely perform cancer treatment with a relatively small dose compared to viral vectors which were no intrinsic oncolytic effect. Therefore, NDV virus or tumor suppressor gene containing recombinant NDV has many advantages in effective treatment method to glioblastoma as a virotherapeutics.

rNDV has an intrinsic oncolytic effect but this study show rNDV-PTEN has more strong oncolytic effect against glioblastoma cells and T98G cell-induced glioblastoma tumor tissue. PTEN gene expression by rNDV-PTEN produced increased anti-tumor effects by mediating cell death and PTEN-mediated apoptosis. In glioblastoma cells treated with rNDV-PTEN, anti-apoptotic protein expression was strongly increased, suggesting that the apoptosis-resistance in glioblastoma had been decreased. NDV has been previously used for delivery of several genes [19, 48–52]. In addition, it has been confirmed that these rNDV-PTEN viruses have intact PTEN CDS even after 10 passages of subculture (Additional file 1: S3,S4). In conclusion, this study shows that rNDV-PTEN has a beneficial effect on the NDV oncolytic function by expressing PTEN in treated glioblastoma.

Currently, several researches used foreign gene insertion site using between P and M genes (Position “2”). This site was known not seriously affect recombinant NDV growth and stable expression of an inserted gene and showed a good effect on cancer suppression but until now any kind of direct comparison test between P and M site (Position “2”) with other four more possible foreign gene insertion sites [53]. In this study, the PTEN gene was inserted between NP and P gene (Position “1”) which position is not used before inserting anticancer gene to make oncolytic virus using NDV. Real-time qPCR and Western blotting results show PTEN gene transcription level by PTEN inserted NDV Position “1” is higher than by PTEN inserted rNDV Position “2”. Because of higher PTEN gene expression of PTEN inserted rNDV Position “1” than of rNDV Position “2”, the oncolytic effect of PTEN inserted rNDV Position “1” also better than the oncolytic effect of PTEN inserted rNDV Position “2”. It means NDV has six major structural genes each gene post-infection in the host cell and mRNA transcription level depends on the distance from the 3′ end of the NDV genome and NP gene mRNA transcription level highest compares with other structural genes. Because of this mechanism of NDV, the closer the foreign gene is inserted to the NP gene, the higher the transcription level of foreign gene mRNA. But still is not clear that mRNA transcription level is not directly correlated with translation level. In addition, further researches are needed to determine whether the difference in inserted foreign gene expression level is dependent on the insertion site or the type of the inserted foreign gene or whether there are other factors.

## Conclusions

In this study show PTEN gene enhance glioblastoma cell killing effect of recombinant NDV, which is one of an intrinsic oncolytic virus. NDV known as a virus pass the brain blood barrier, therefore recombinant NDV containing cancer suppressor genes will be good candidate for virotherapeutics for brain cancer treatment.

## Supplementary Information

**Additional file 1:** The results of rNDV-PTEN sequencing, quantification of western blotting, and orthotropic glioblastoma model in vivo test.

## Data Availability

All data generated or analyzed during this study are included in this published article.

## Abbreviations

PTEN: Phosphatase and tensin homolog
NDV: Newcastle disease virus
GBM: Glioblastoma
BBB: Brain-blood barrier
