# Supplementary material for: The phosphatase and tensin homolog gene inserted between NP and P gene of recombinant New castle disease virus oncolytic effect test to glioblastoma cell and xenograft mouse model
Source: Virol J. 2022 Jan 29;19:21. doi: 10.1186/s12985-022-01746-w (PMC8800283; doi:10.1186/s12985-022-01746-w)
Supplement: Supplementary file 1 — Additional file 1: The results of rNDV-PTEN sequencing, quantification of western blotting, and orthotropic glioblastoma model in vivo test. [file 12985_2022_1746_MOESM1_ESM.pdf]

Supplementary Material

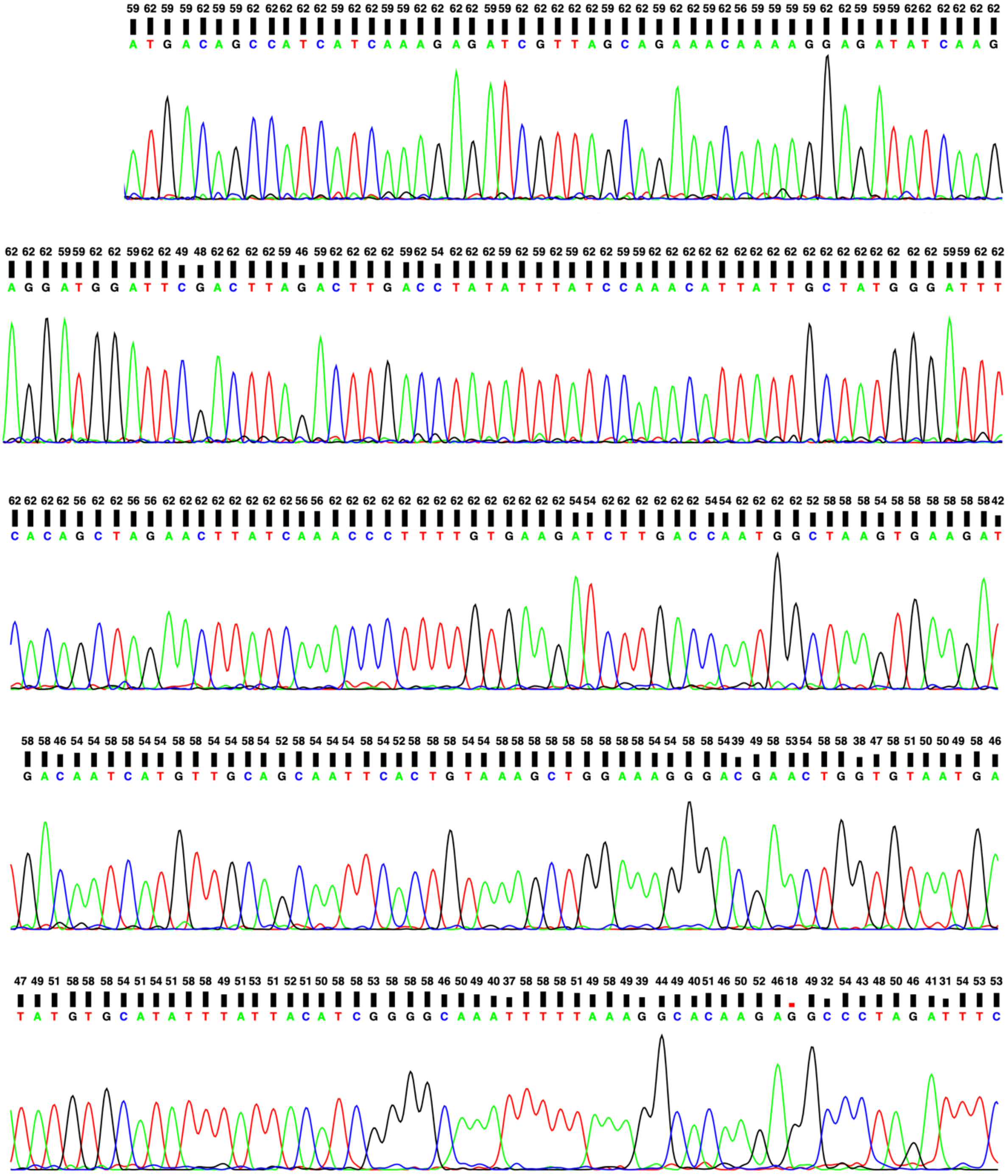

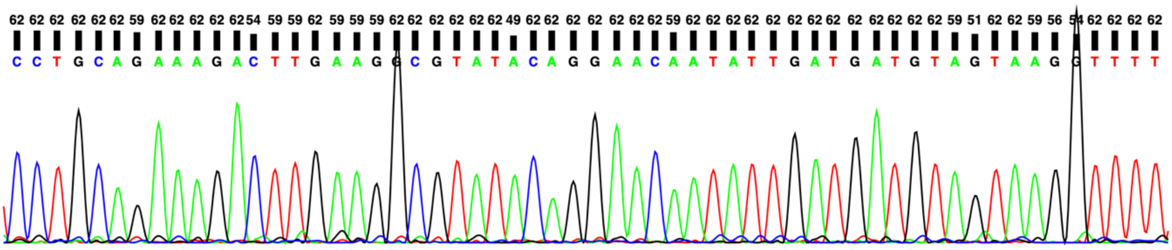
C C T G C A G A A A G A C T T G A A G G C G T A T A C A G G A A C A A T A T T G A T G A T G T A G T A A G G T T T T

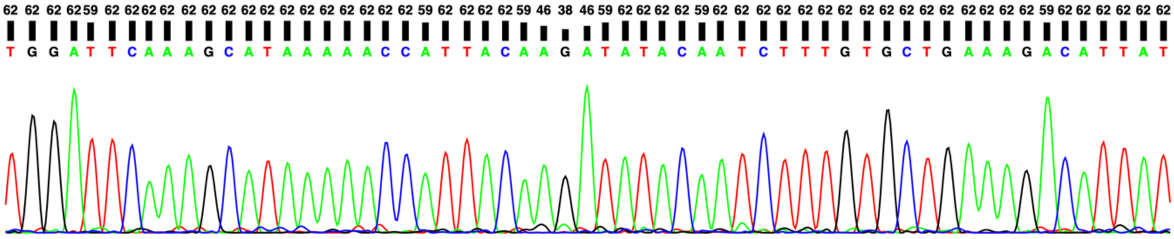
T G G A T T C A A A G C A T A A A A A C C A T T A C A A G A T A T A C A A T C T T T G T G C T G A A A G A C A T T A T

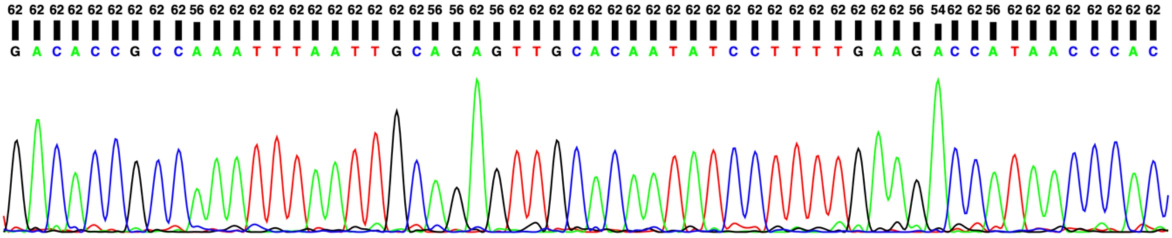
G A C A C C G C C A A A T T T A A T T G C A G A G T T G C A C A A T A T C C T T T T G A A G A C C A T A A C C C A C

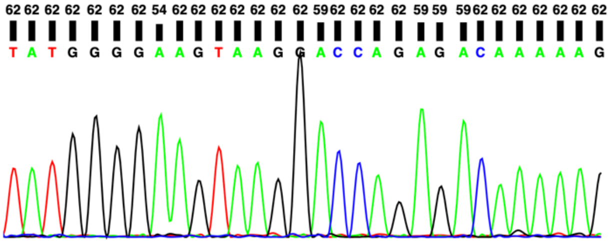
T A T G G G G A A G T A A G G A C C A G A G A C A A A A A G

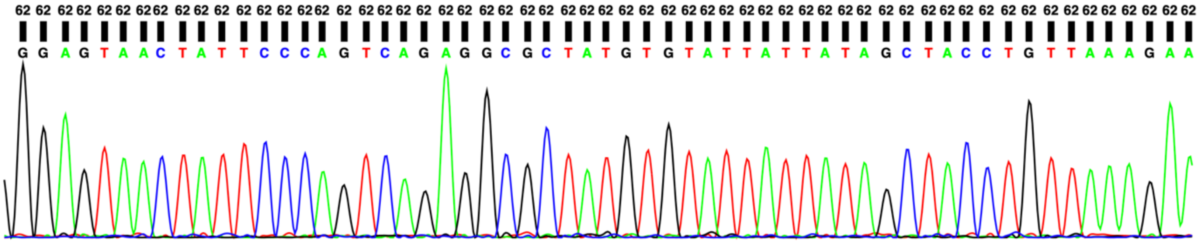
G G A G T A A C T A T T C C C A G T C A G A G G C G C T A T G T G T A T T A T T A T A G C T A C C T G T T A A A G A A

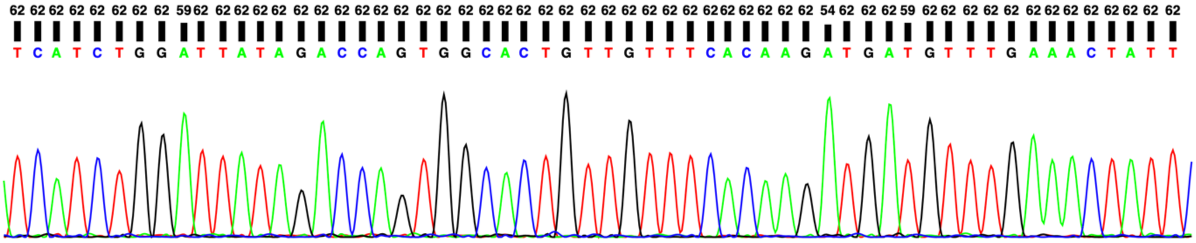
T C A T C T G G A T T A T A G A C C A G T G G C A C T G T T G T T T C A C A A G A T G A T G T T T G A A A C T A T T

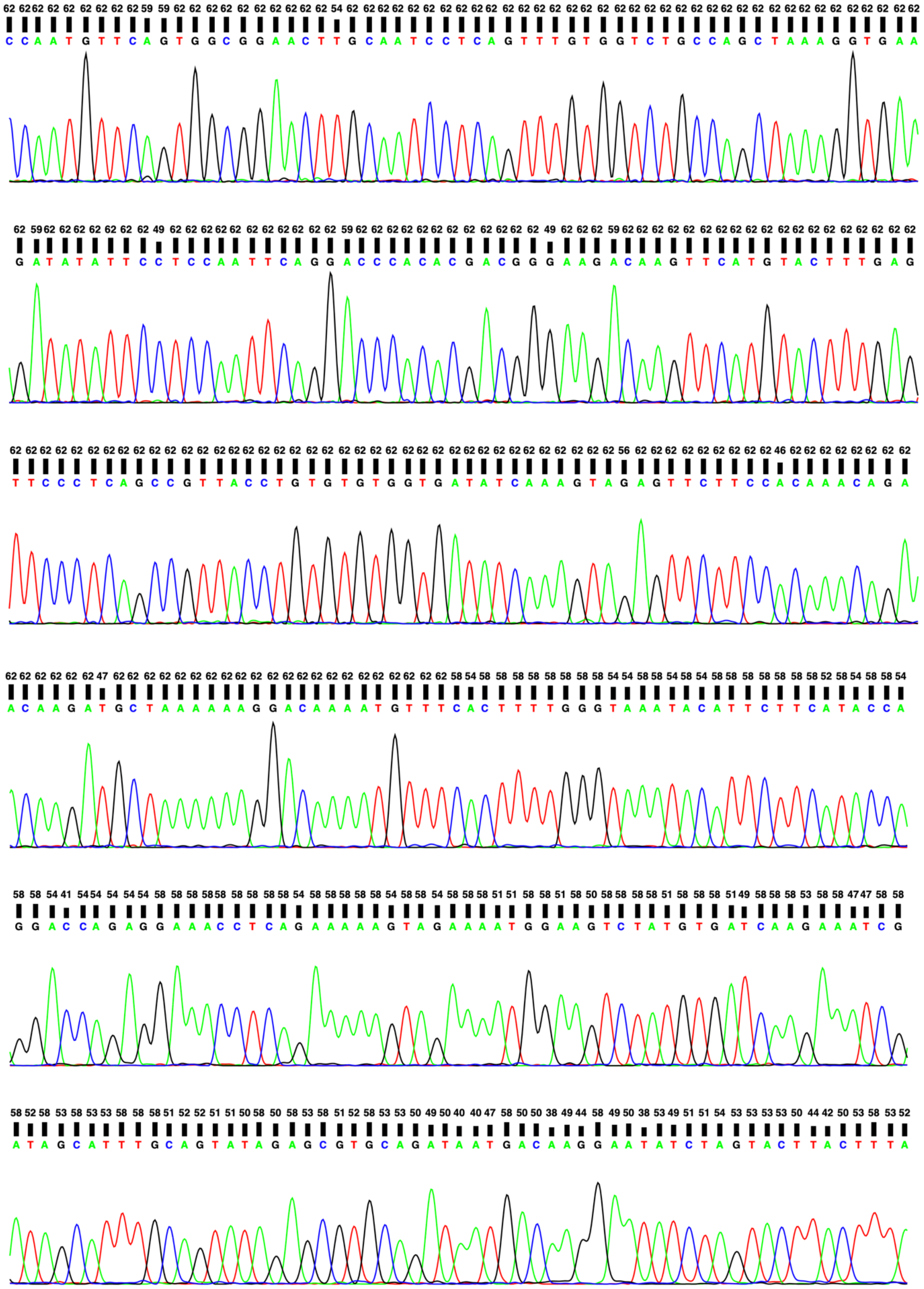
C C A A T G T T C A G T G G C G G A A C T T G C A A T C C T C A G T T T G T G G T C T G C C A G C T A A A G G T G A A
G A T A T A T T C C T C C A A T T C A G G A C C C A C A C G A C G G G A A G A C A A G T T C A T G T A C T T T G A G
T T C C C T C A G C C G T T A C C T G T G T G T G G T G A T A T C A A A G T A G A G T T C T T C C A C A A A C A G A
A C A A G A T G C T A A A A A A G G A C A A A A T G T T T C A C T T T T G G G T A A A T A C A T T C T T C A T A C C A
G G A C C A G A G G A A A C C T C A G A A A A A G T A G A A A A T G G A A G T C T A T G T G A T C A A G A A A T C G
A T A G C A T T T G C A G T A T A G A G C G T G C A G A T A A T G A C A A G G A A T A T C T A G T A C T T A C T T T A

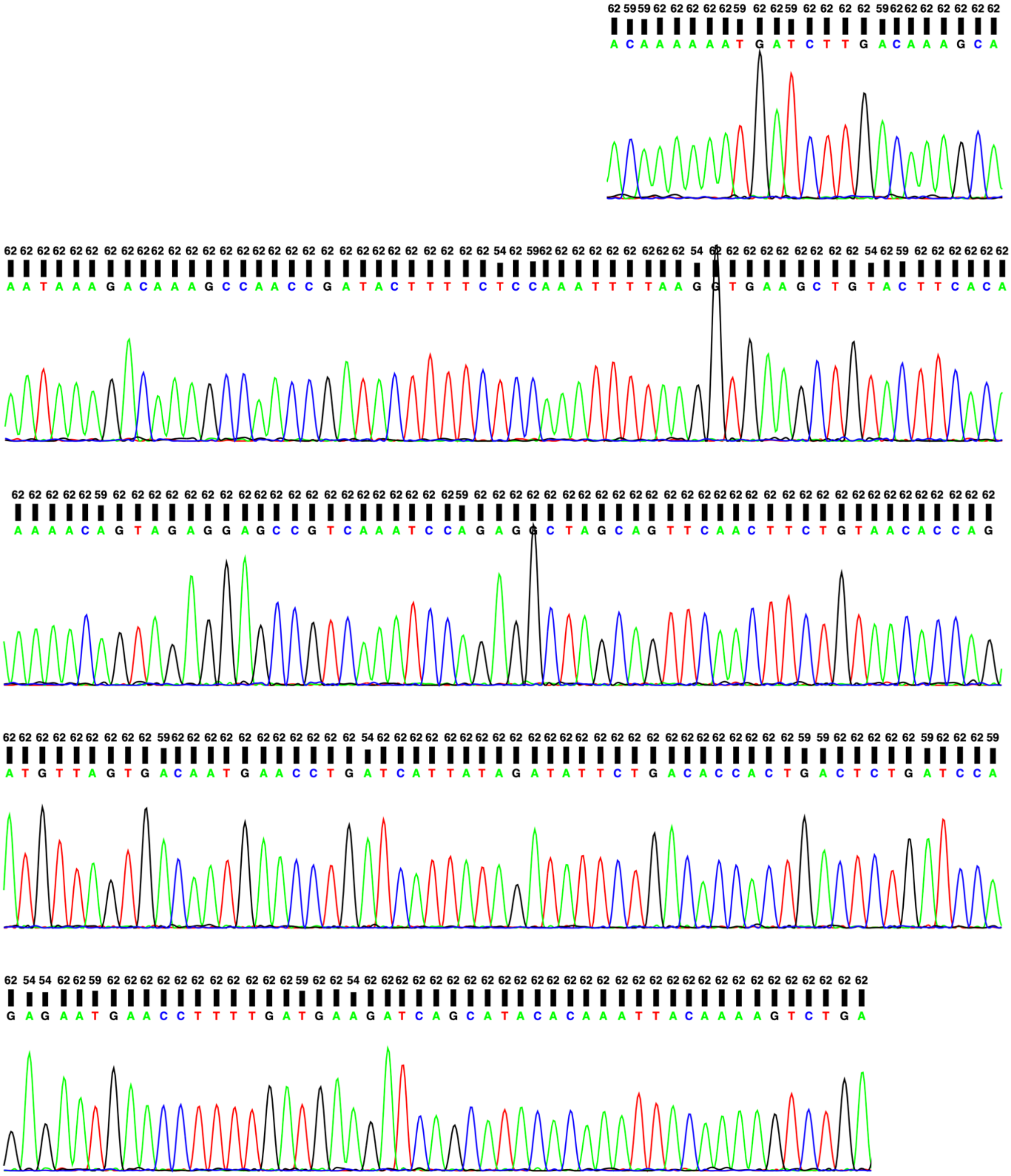

S1. Sequencing result of PTEN CDS in rescued rNDV-PTEN Position"1"

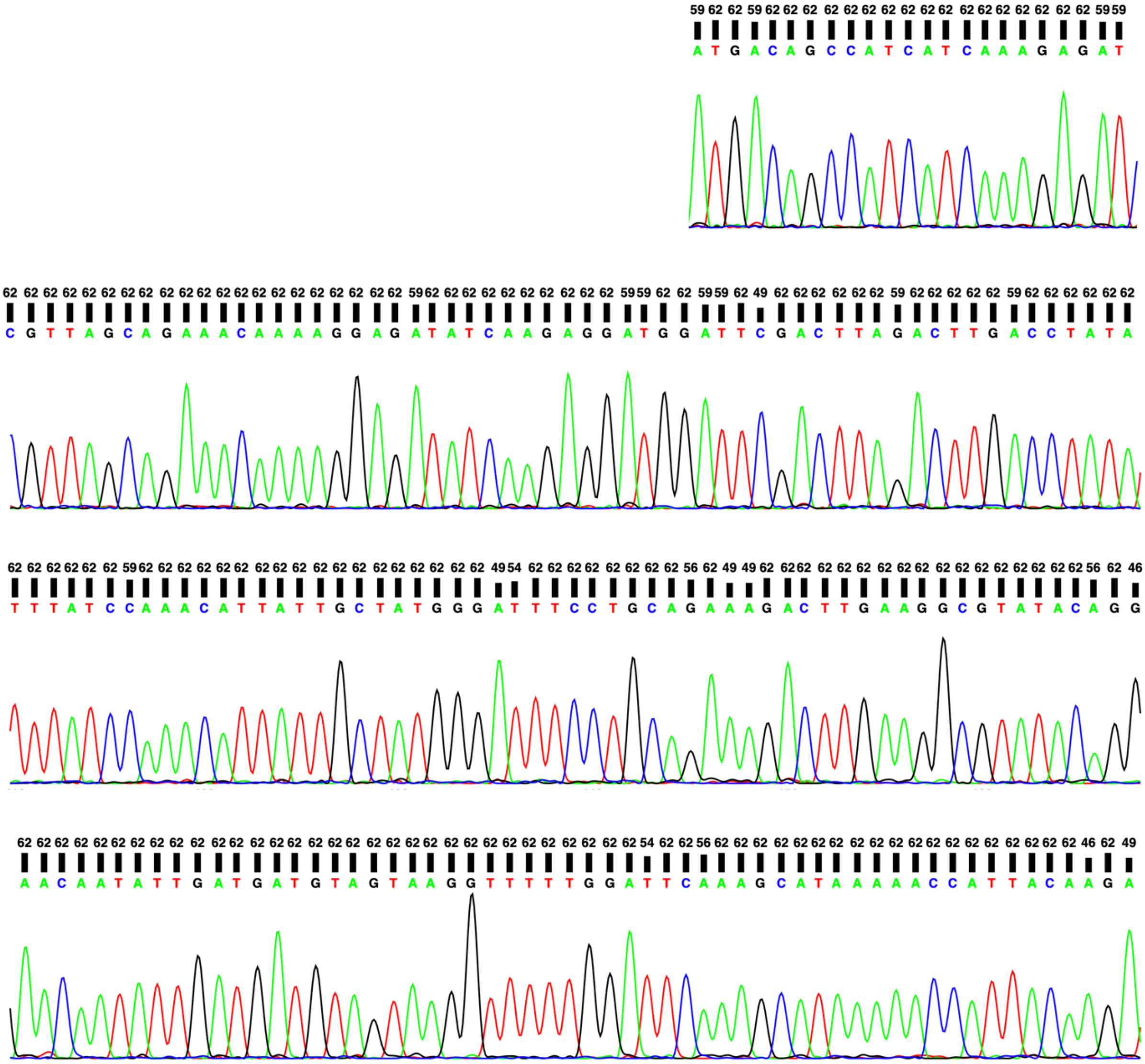
ATGACAGCCATCATCAAAGAGAT
CGTTAGCAGAAACAAAAGGAGATATCAAGAGGATGGATTCGACTTAGACTTGACCTATA
TTTATCCAAACATTATTGCTATGGGATTTCCTGCAGAAAGACTTGAAGGCGTATACAGG
AACAATATTGATGATGTAGTAAGGTTTTTGGATTCAAAGCATAAAAACCATTACAAGA

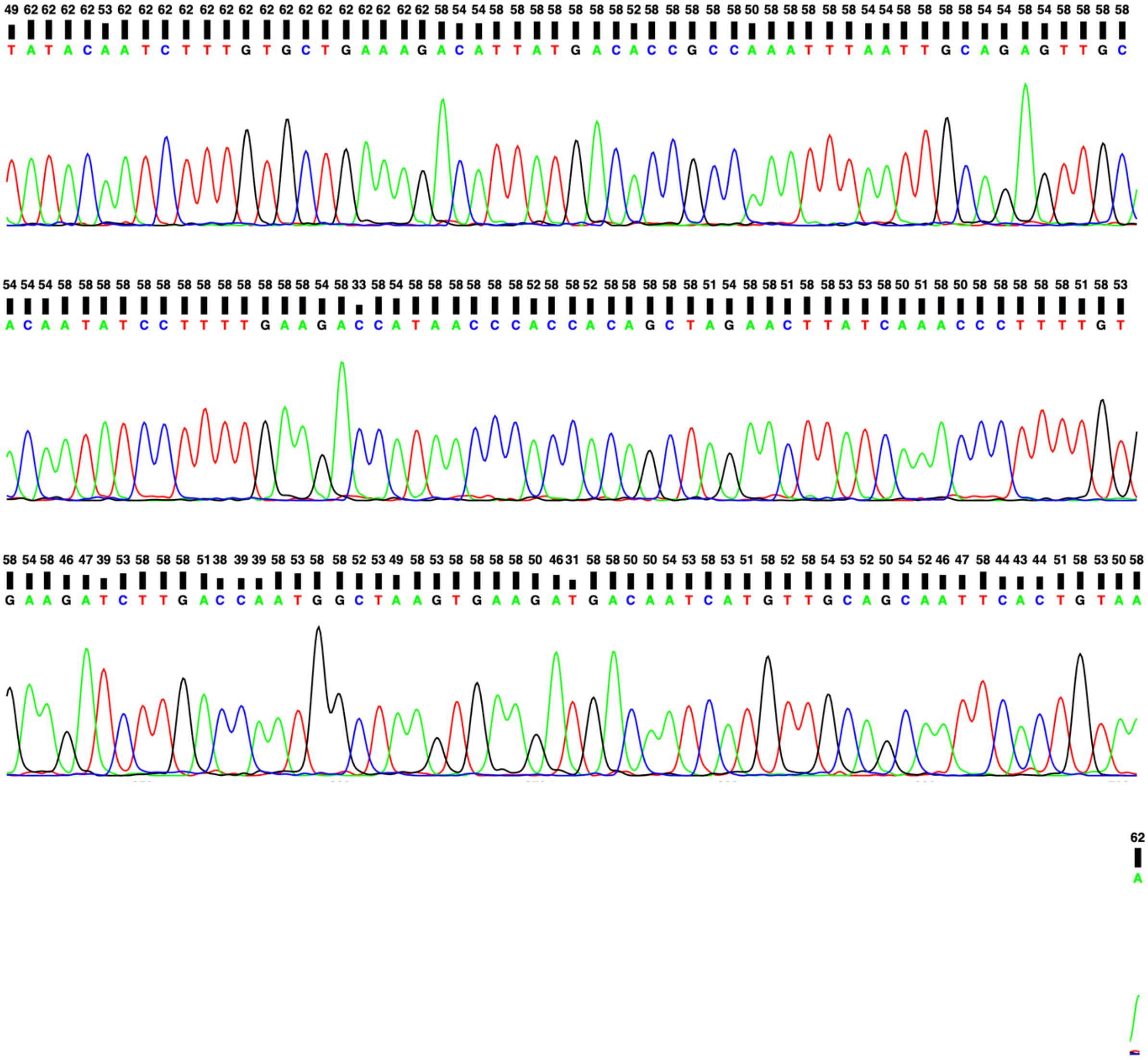

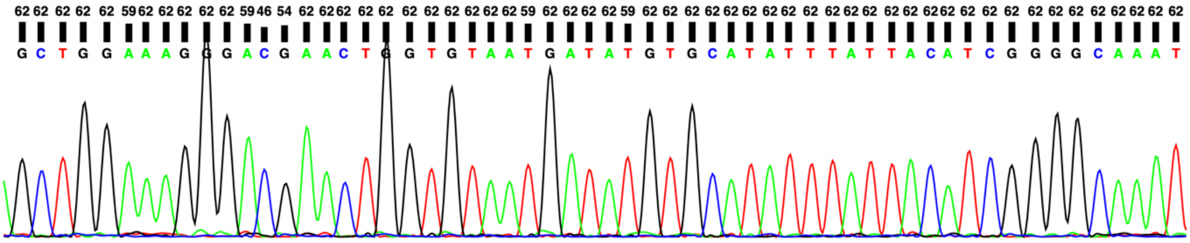

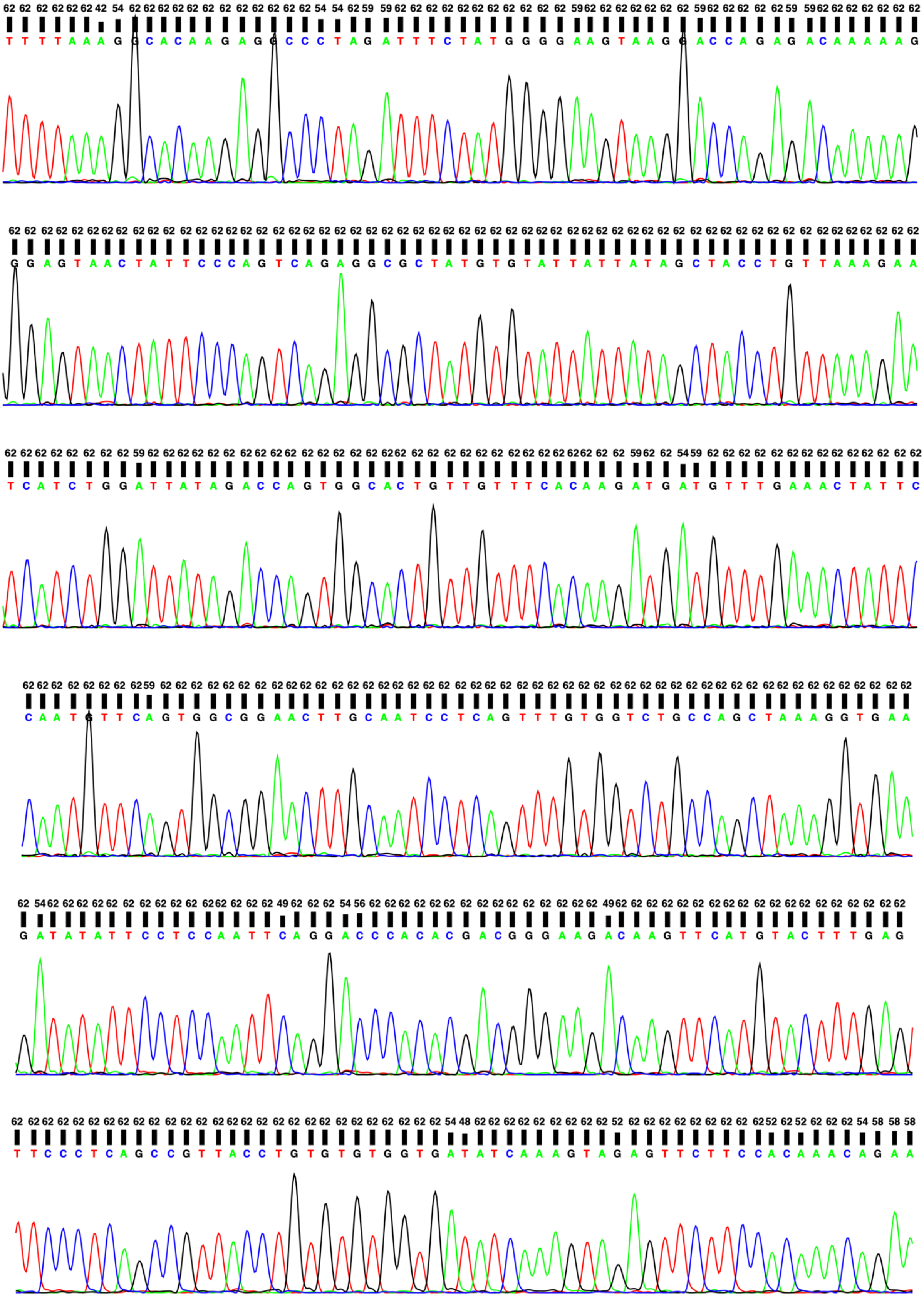
T T T T A A A G G C A C A A G A G G C C C T A G A T T T C T A T G G G G A A G T A A G G A C C A G A G A C A A A A A G
G G A G T A A C T A T T C C C A G T C A G A G G C G C T A T G T G T A T T A T T A T A G C T A C C T G T T A A A G A A
T C A T C T G G A T T A T A G A C C A G T G G C A C T G T T G T T T C A C A A G A T G A T G T T T G A A A C T A T T C
C A A T G T T C A G T G G C G G A A C T T G C A A T C C T C A G T T T G T G G T C T G C C A G C T A A A G G T G A A
G A T A T A T T C C T C C A A T T C A G G A C C C A C A C G A C G G G A A G A C A A G T T C A T G T A C T T T G A G
T T C C C T C A G C C G T T A C C T G T G T G T G G T G A T A T C A A A G T A G A G T T C T T C C A C A A A C A G A A

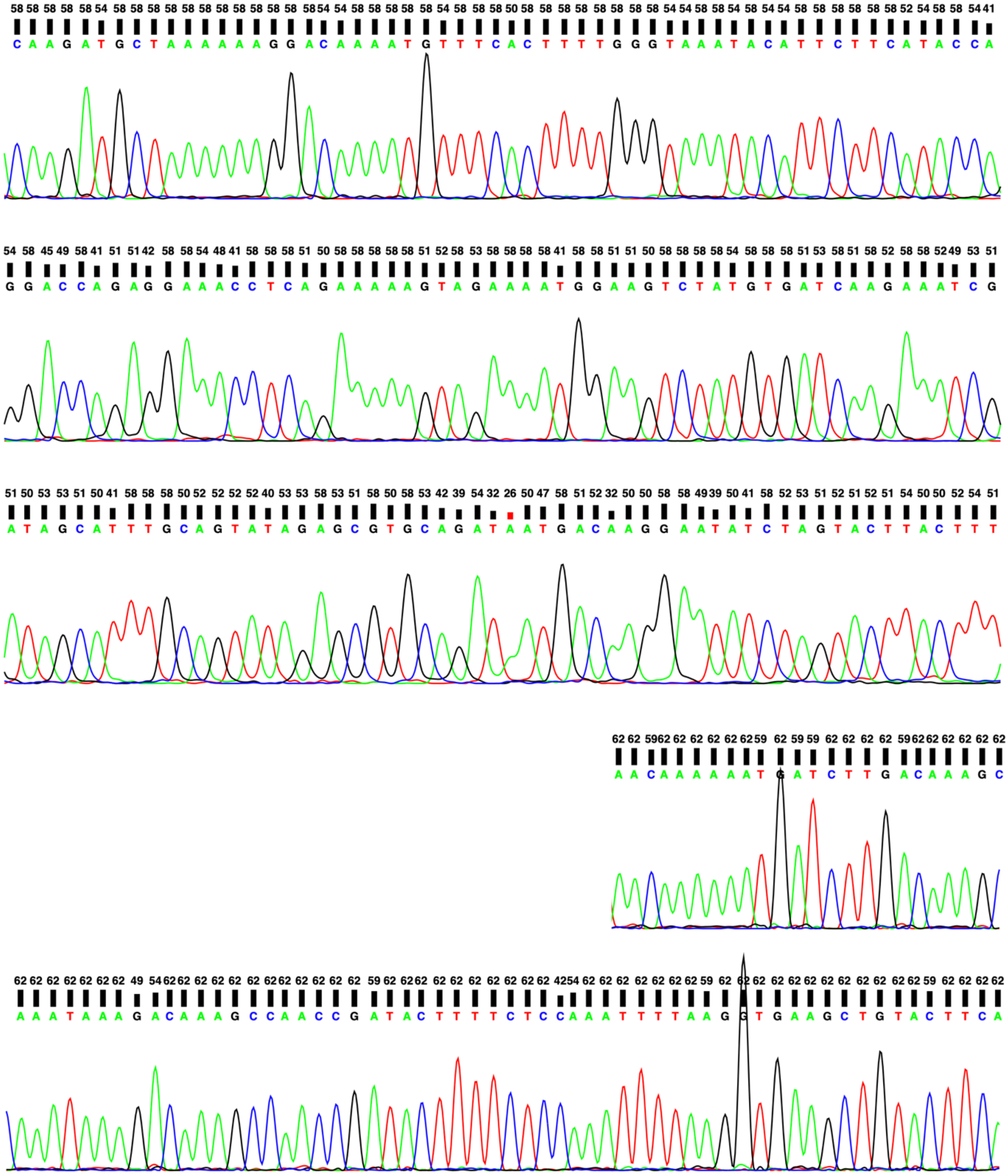
C A A G A T G C T A A A A A A G G A C A A A A T G T T T C A C T T T T G G G T A A A T A C A T T C T T C A T A C C A
G G A C C A G A G G A A A C C T C A G A A A A A G T A G A A A A T G G A A G T C T A T G T G A T C A A G A A A T C G
A T A G C A T T T G C A G T A T A G A G C G T G C A G A T A A T G A C A A G G A A T A T C T A G T A C T T A C T T T
A A C A A A A A T G A T C T T G A C A A A G C
A A A T A A A G A C A A A G C C A A C C G A T A C T T T T C T C C A A A T T T T A A G G T G A A G C T G T A C T T C A

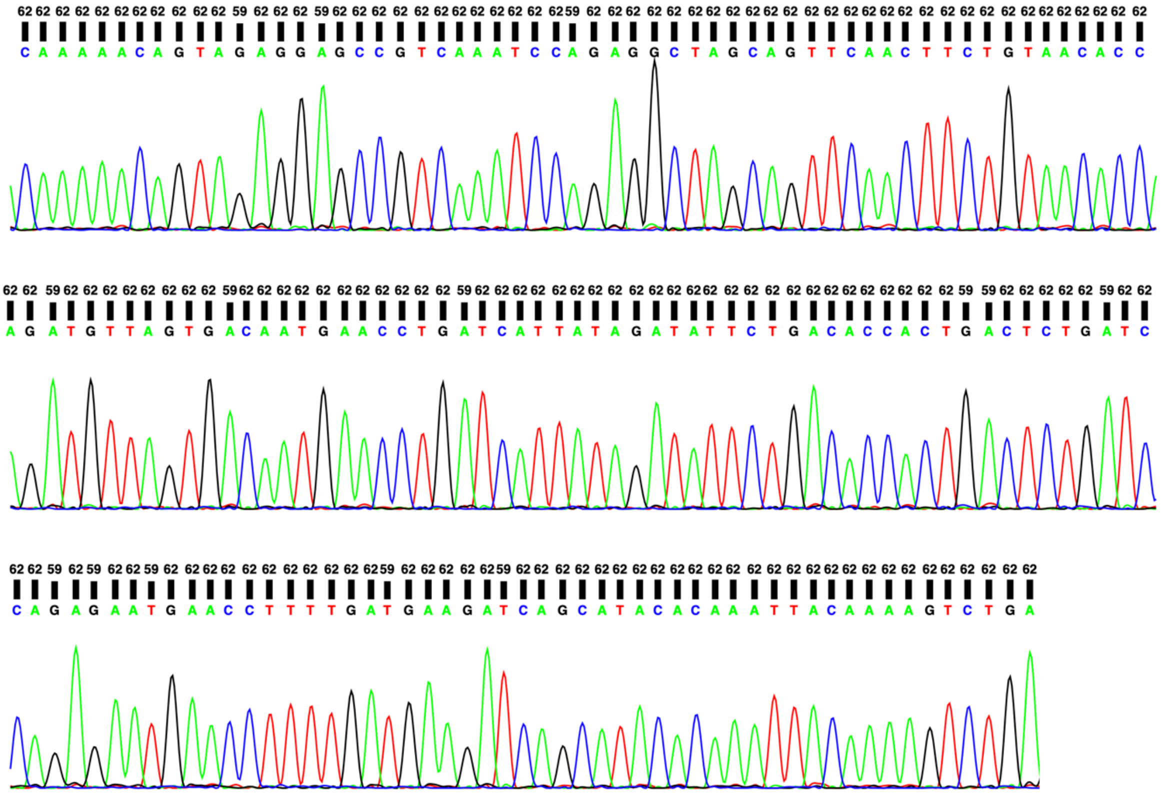

S2. Sequencing result of PTEN CDS in rescued rNDV-PTEN Position"2"

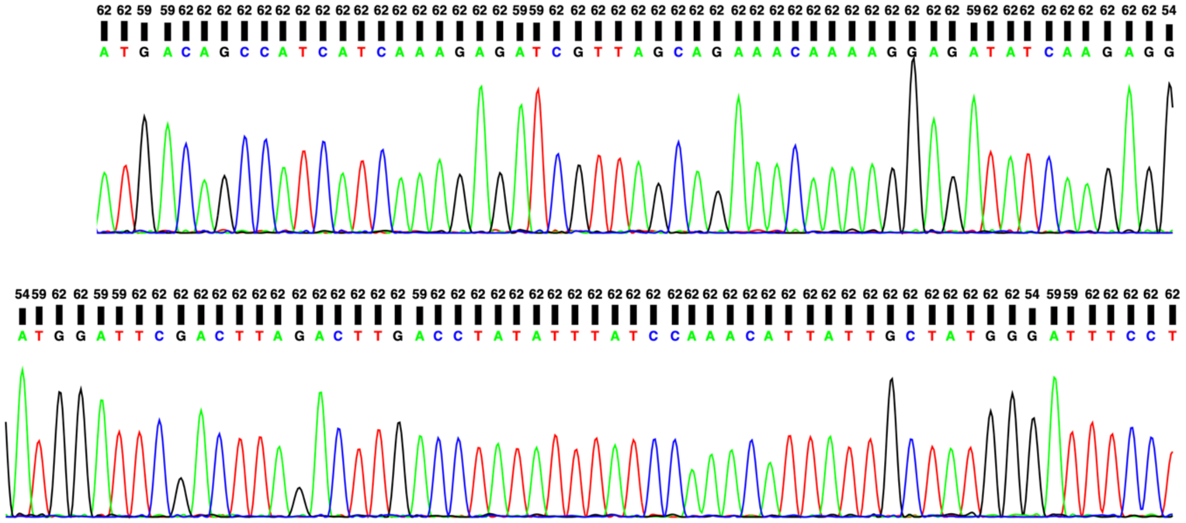

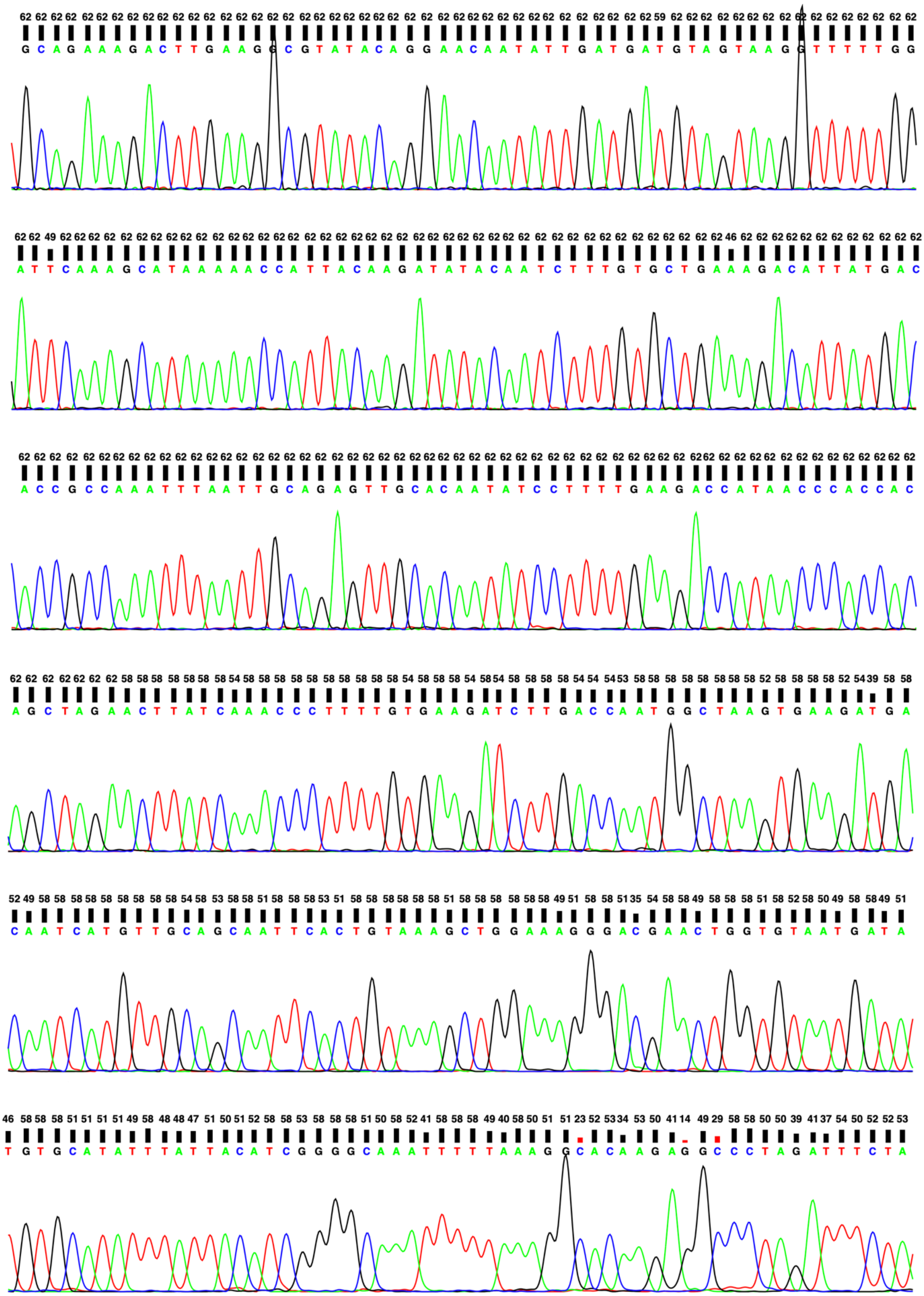

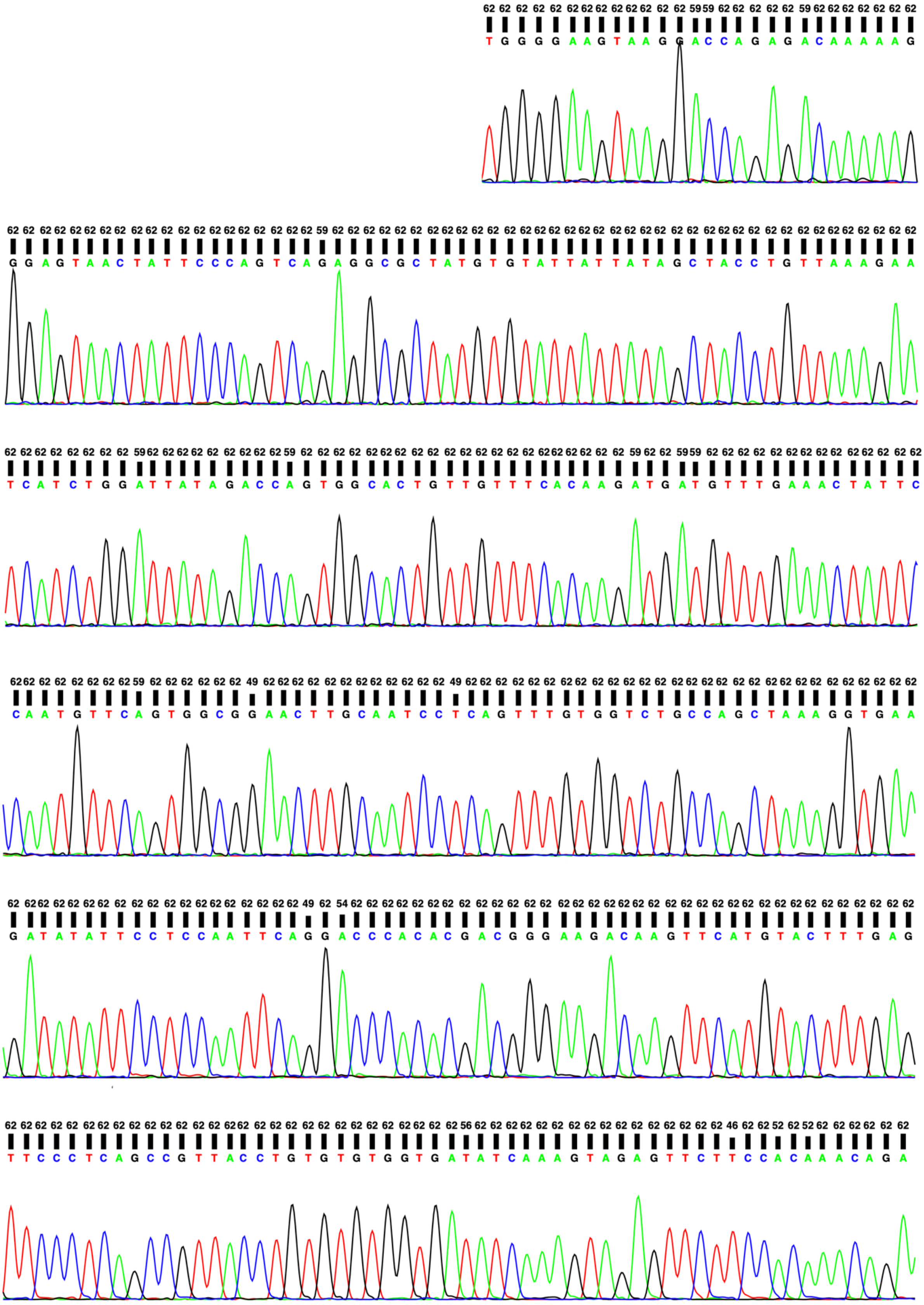
T G G G G A A G T A A G G A C C A G A G A C A A A A A G
G G A G T A A C T A T T C C C A G T C A G A G G C G C T A T G T G T A T T A T T A T A G C T A C C T G T T A A A G A A
T C A T C T G G A T T A T A G A C C A G T G G C A C T G T T G T T T C A C A A G A T G A T G T T T G A A A C T A T T C
C A A T G T T C A G T G G C G G A A C T T G C A A T C C T C A G T T T G T G G T C T G C C A G C T A A A G G T G A A
G A T A T A T T C C T C C A A T T C A G G A C C C A C A C G A C G G G A A G A C A A G T T C A T G T A C T T T G A G
T T C C C T C A G C C G T T A C C T G T G T G T G G T G A T A T C A A A G T A G A G T T C T T C C A C A C A A A C A G A

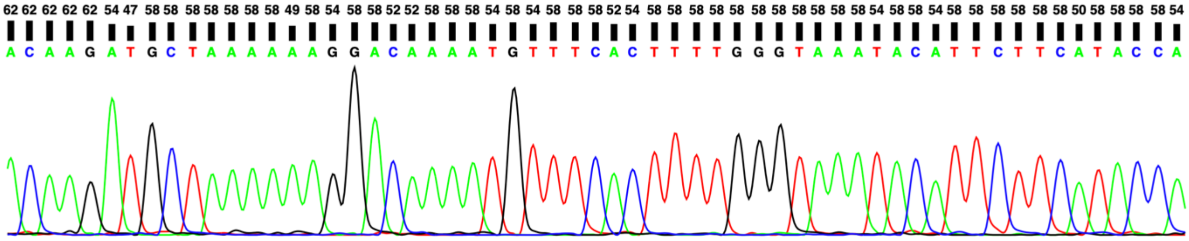
A C A A G A T G C T A A A A A A G G A C A A A A T G T T T C A C T T T T G G G T A A A T A C A T T C T T C A T A C C A

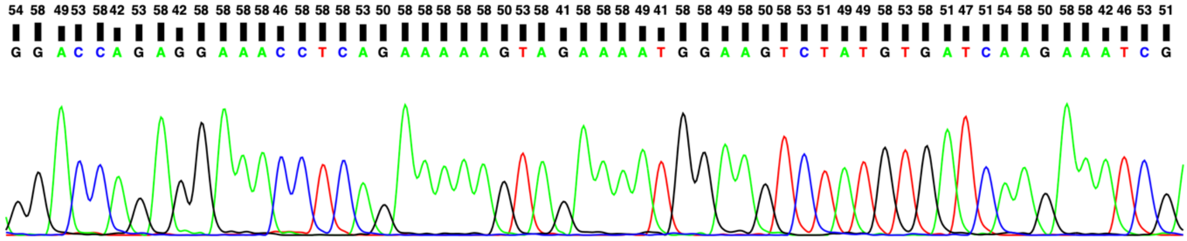
G G A C C A G A G G A A A C C T C A G A A A A A G T A G A A A A T G G A A G T C T A T G T G A T C A A G A A A T C G

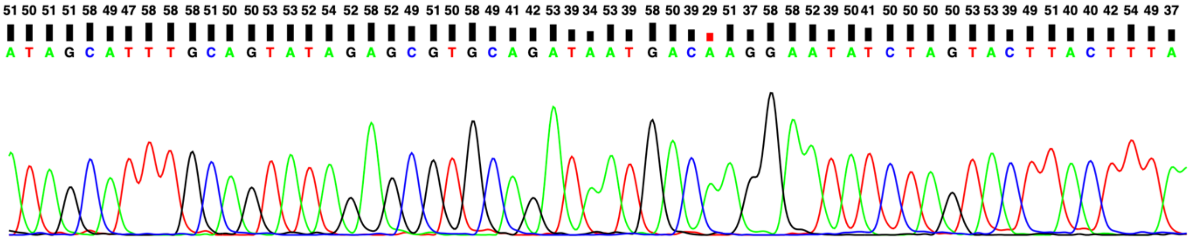
A T A G C A T T T G C A G T A T A G A G C G T G C A G A T A A T G A C A A G G A A T A T C T A G T A C T T A C T T T A

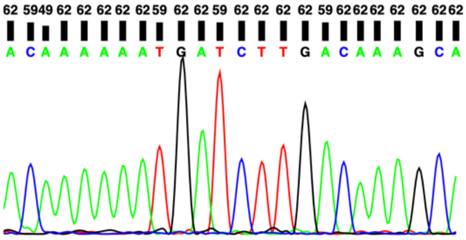
A C A A A A A T G A T C T T G A C A A A G C A

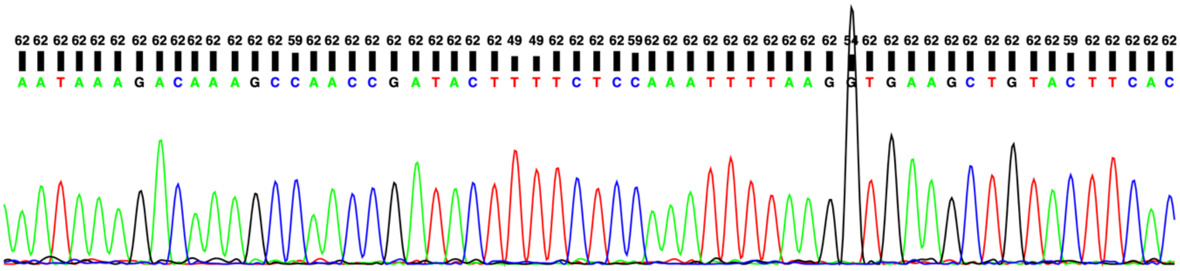
A A T A A A G A C A A A G C C A A C C G A T A C T T T T C T C C A A A T T T T A A G G T G A A G C T G T A C T T C A C

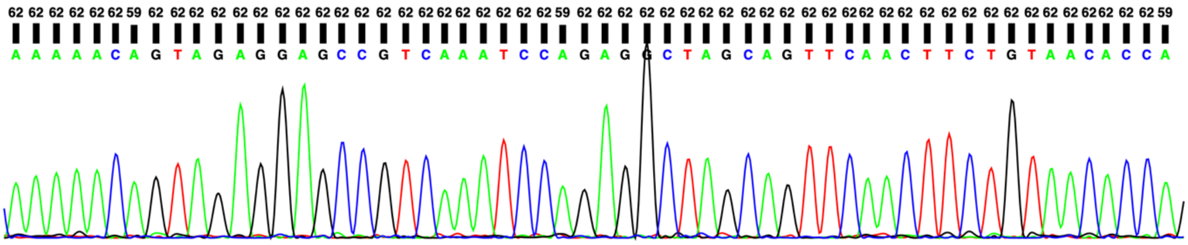

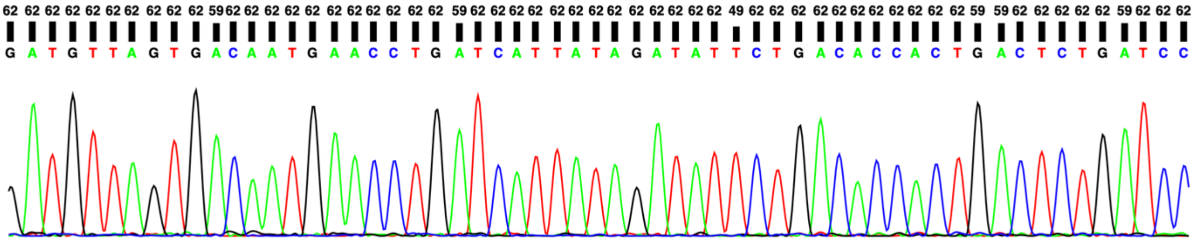

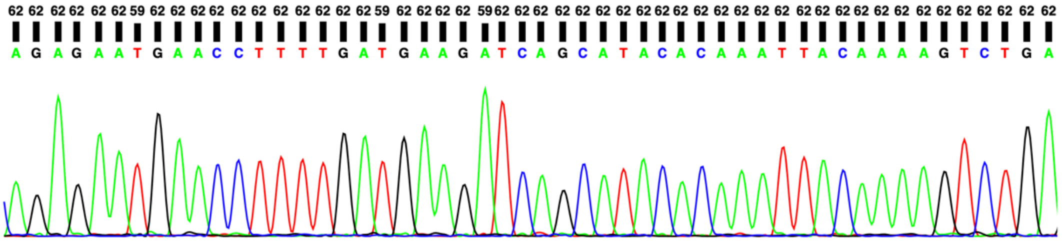

S3. Sequencing result of PTEN CDS in rNDV-PTEN Position”1” Passage.10

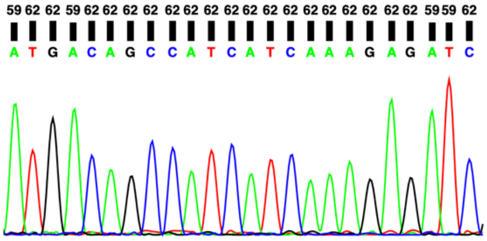

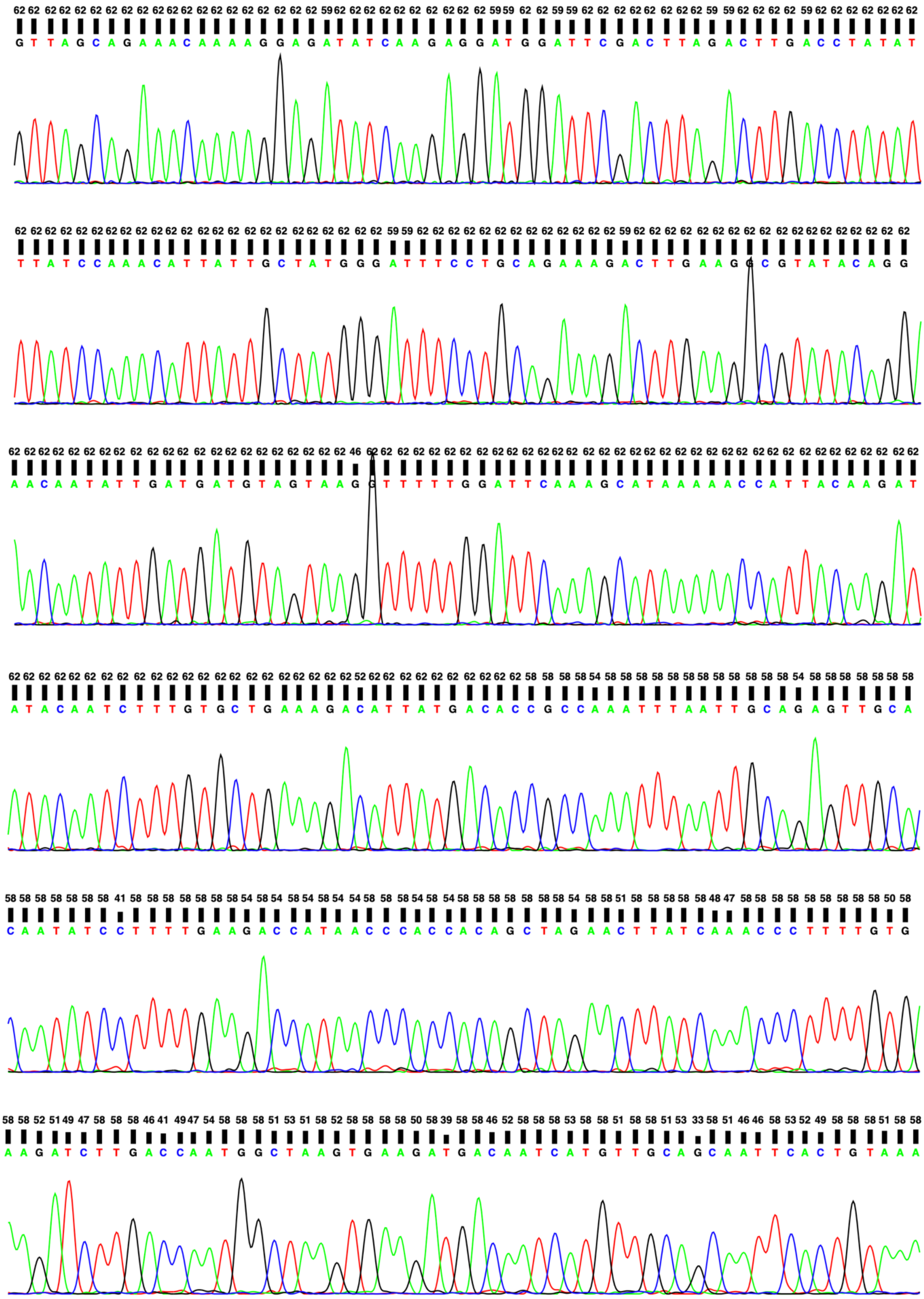

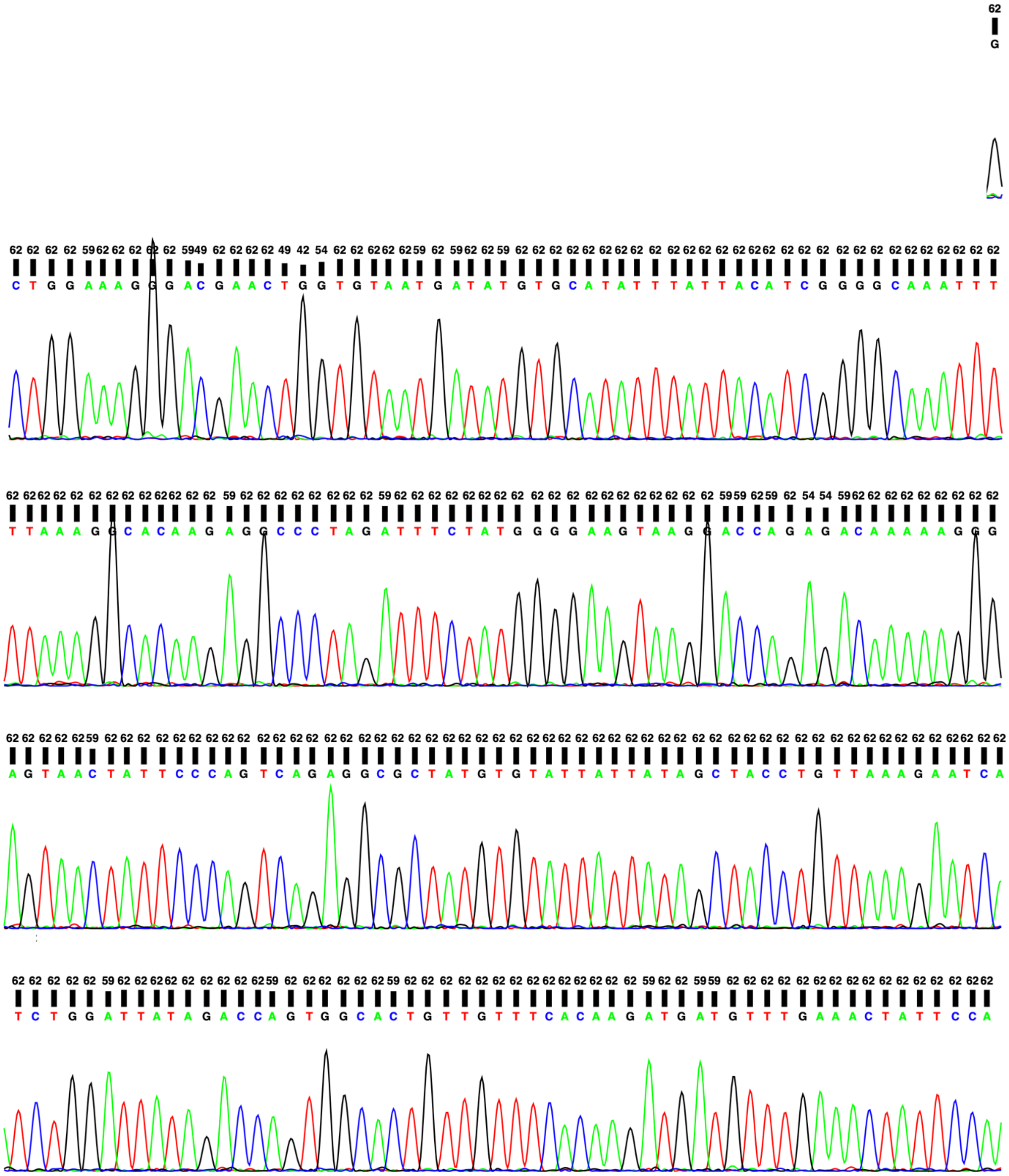
G
CTGGAAAGGGACGAACTGGTGTAATGATATGTGCATATTTATTACATCGGGGCAAATTT
TTAAAGGCACAAGAGGCCCTAGATTTCTATGGGGAAGTAAGGACCAGAGACAAAAAGGG
AGTAACTATTCCCAGTCAGAGGCGCTATGTGTATTATTATAGCTACCTGTTAAAGAATCA
TCTGGATTATAGACCAGTGGCACTGTTGTTTCACAAGATGATGTTTGAAACTATTCCA

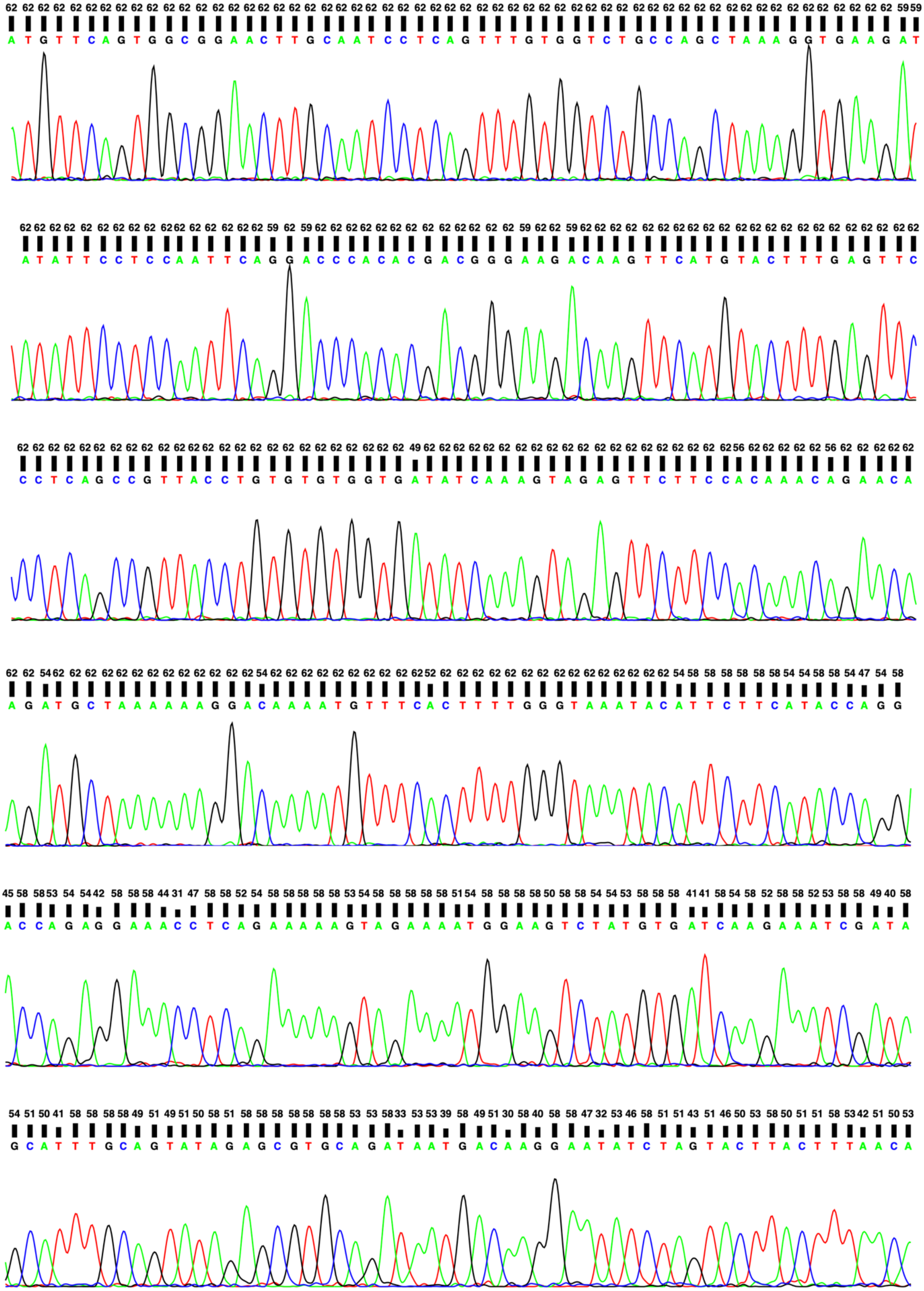

A T G T T C A G T G G C G G A A C T T G C A A T C C T C A G T T T G T G G T C T G C C A G C T A A A G G T G A A G A T
A T A T T C C T C C A A T T C A G G A C C C A C A C G A C G G G A A G A C A A G T T C A T G T A C T T T G A G T T C
C C T C A G C C G T T A C C T G T G T G T G G T G A T A T C A A A G T A G A G T T C T T C C A C A A A C A G A A C A
A G A T G C T A A A A A A G G A C A A A A T G T T T C A C T T T T G G G T A A A T A C A T T C T T C A T A C C A G G
A C C A G A G G A A A C C T C A G A A A A A G T A G A A A A T G G A A G T C T A T G T G A T C A A G A A A T C G A T A
G C A T T T G C A G T A T A G A G C G T G C A G A T A A T G A C A A G G A A T A T C T A G T A C T T A C T T T A A C A

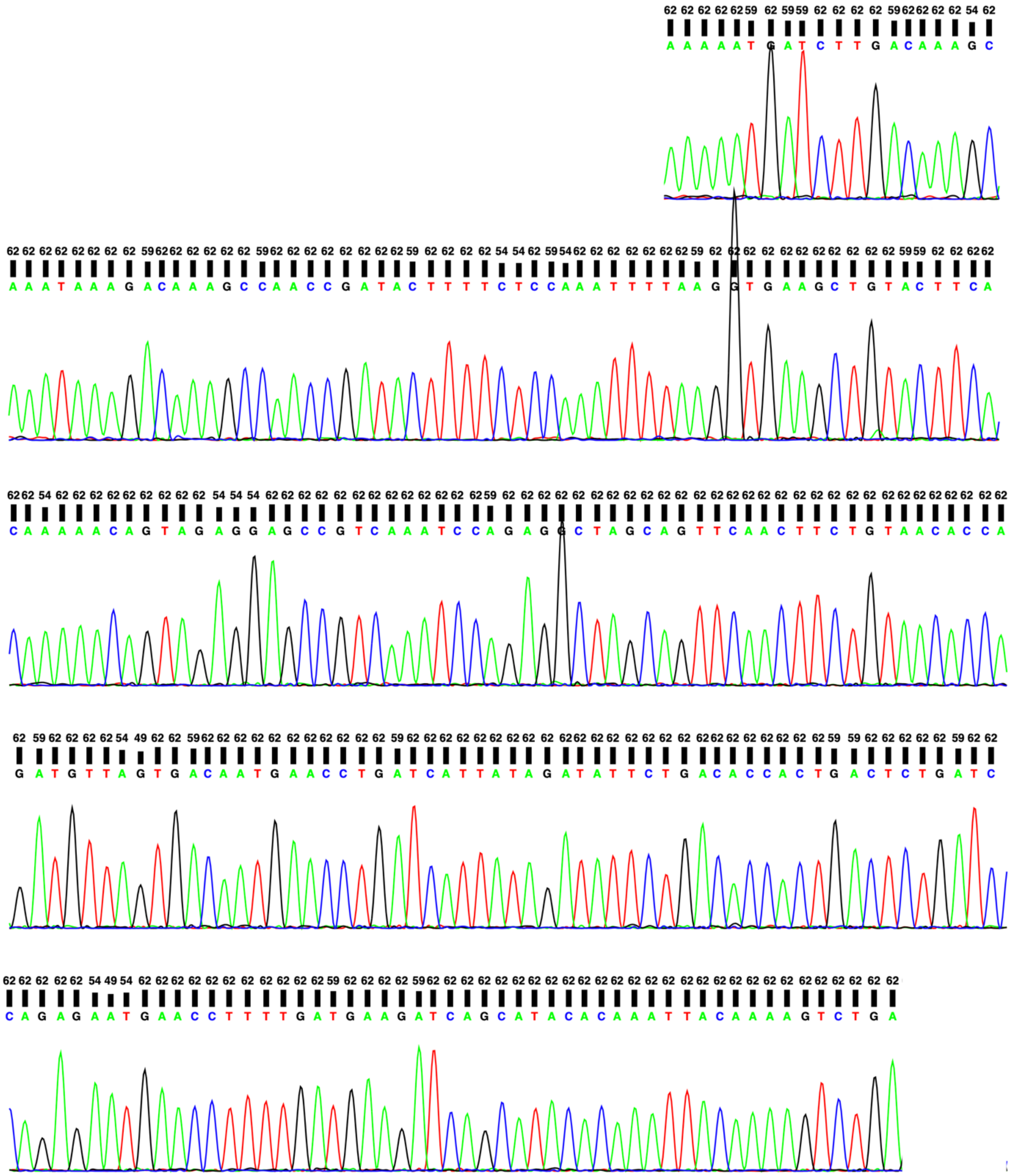

S4. Sequencing result of PTEN CDS in rNDV-PTEN Position"2" Passage.10

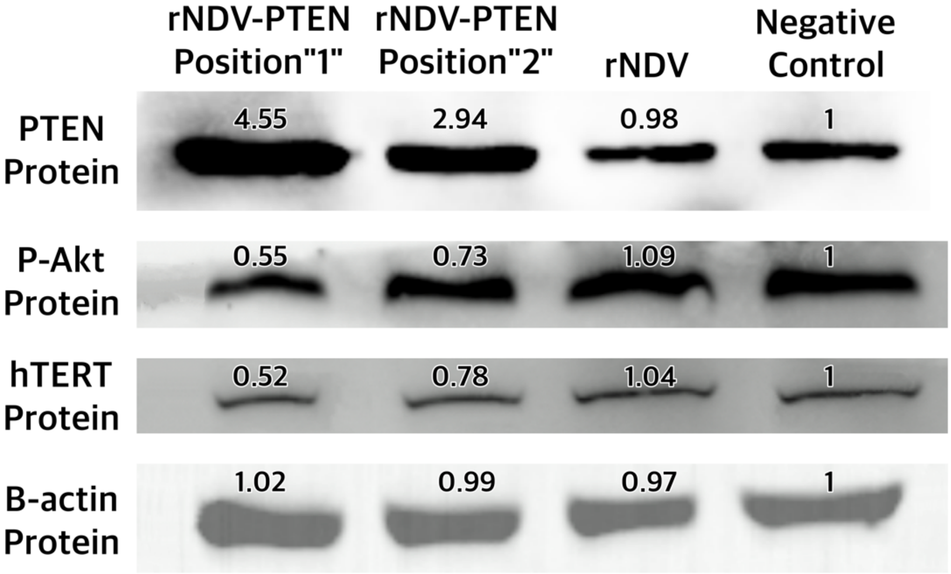

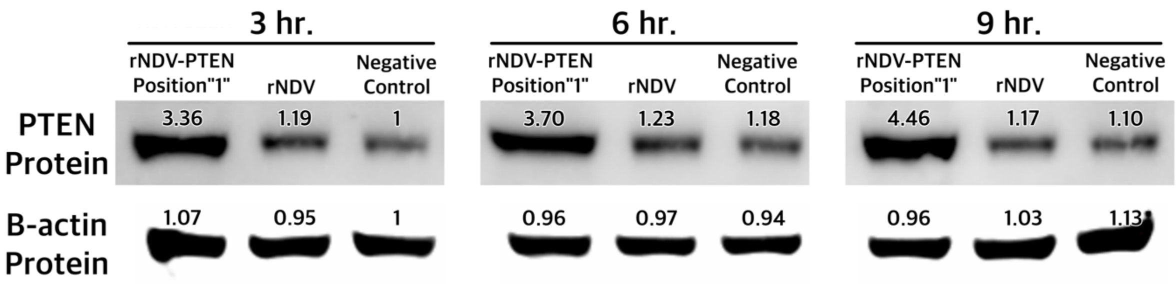

S5. Quantification of Results of Western blotting

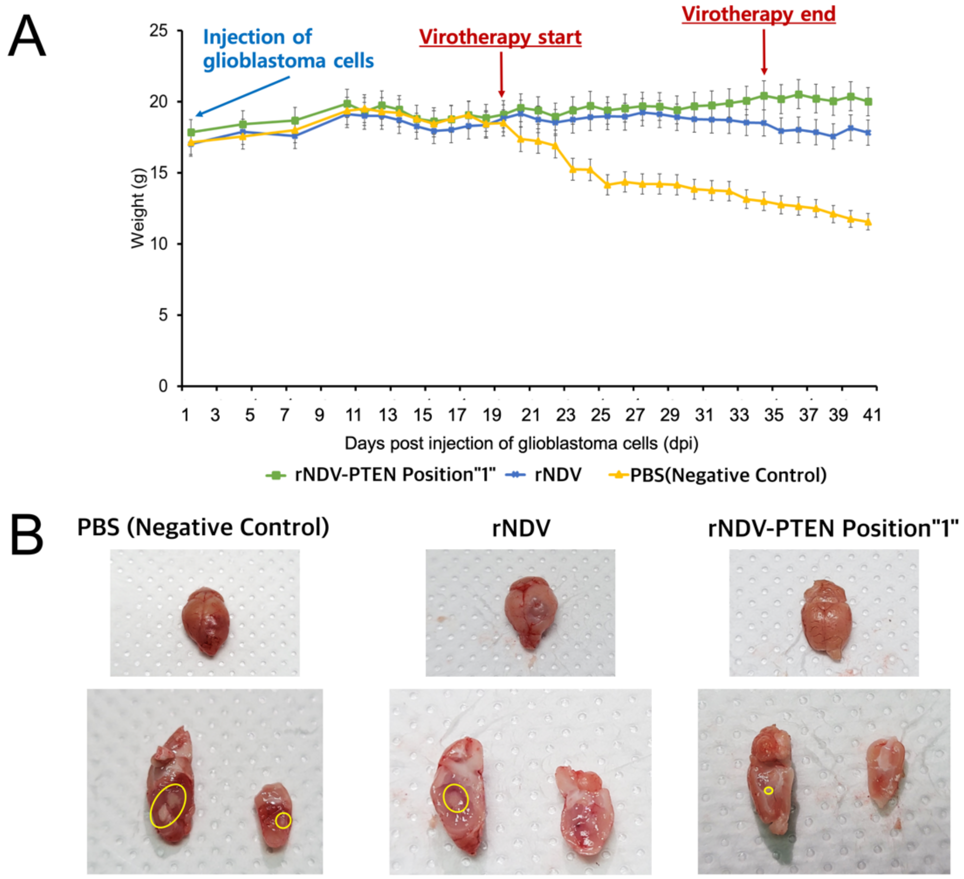

S6. orthotropic glioblastoma model in vivo test. A)Body weight graph of PTEN inserted rNDV Position “1”, rNDV and PBS(negative control) inoculated mice. B)A cross-sectional photograph of PTEN inserted rNDV Position “1”, rNDV and PBS(negative control) inoculated orthotopic glioblastoma model brain. Yellow circle indicates tumor.
